# Integrated analysis of transcriptome, sRNAome and degradome sequencing provides insights into bacterial wilt resistance in potato

**DOI:** 10.1186/s12870-025-07768-0

**Published:** 2025-12-05

**Authors:** Yu Yang, Xiaoyuan Zhang, Jiaping Ma, Jun Xiao, Zhengxiang Feng, Junmei Yu, Wanjie Li, Pengfei Jiang, Guangtao Zhu, Yupeng Geng, Junzhong Liu

**Affiliations:** 1 Yunnan Key Laboratory of Cell Metabolism and Diseases, Center for Life Science and School of Life Sciences, Yunnan University, Kunming, 650500 China; 2 School of Life Sciences, The National Engineering Laboratory of Crop Resistance Breeding, Anhui Agricultural University, Hefei, 230036 China; 3 School of Life Sciences, Yunnan Key Laboratory of Potato Biology, Yunnan Normal University, Southwest United Graduate School, Kunming, 650500 China; 4 State Key Laboratory for Vegetation Structure, Function and Construction (VegLab), Ministry of Education Key Laboratory for Transboundary Ecosecurity of Southwest China, School of Ecology and Environmental Science, Yunnan University, Kunming, 650500 China

**Keywords:** Bacterial wilt, Small RNAs, Degradome, Transcriptome, Potato

## Abstract

**Background:**

Potato (*Solanum tuberosum*) is one of the most important global food crops. However, potato bacterial wilt, a destructive soil-borne disease caused by *Ralstonia solanacearum*, poses a huge threat to global potato production and quality, leading to serious economic losses worldwide. The wild potato species *Solanum commersonii* exhibits resistance to bacterial wilt, but the underlying molecular mechanisms remain largely obscure.

**Results:**

In this study, we identified differentially expressed miRNAs (microRNAs), phased secondary short-interfering RNAs (phasiRNAs), along with their target transcripts, and elucidated the potential pathways involved in bacterial wilt resistance through high-throughput sequencing analyses of transcriptome, sRNAome, and degradome on normal and *R. solanacearum*-infected potato roots, which were collected from the susceptible diploid potato clone *S. tuberosum* group Phureja SP15-65 and the resistant diploid *S. commersonii* germplasm CM804. The results revealed that 3,434 and 1,045 differentially expressed genes (DEGs) were identified in susceptible SP15-65 and resistant CM804, respectively, with 7,652 DEGs identified between SP15-65 and CM804 upon pathogen inoculation. 23 transcripts specifically expressed in CM804 were identified to be responsive to *R. solanacearum* infection. Functional enrichment analysis of DEGs revealed that mitogen-activated protein kinase (MAPK) activation, reactive oxygen species (ROS) generation, calcium signaling, hormone signaling, secondary metabolism, and transcriptional reprogramming for defense were potential pathways in potato root response to *R. solanacearum* infection. Furthermore, 115 unique known and 147 putative novel miRNAs showed differential expression in SP15-65 or CM804 after pathogen infection. Among these differentially expressed miRNAs, some *miR482*, *miR6024*, and *miR390* family members triggered the biosynthesis of phasiRNAs through the cleavage of phasiRNA-generating (*PHAS*) precursor transcripts, as validated by degradome-seq. The resulting phasiRNAs directed the cleavage of their downstream target mRNAs. Six miRNA-mRNA pairs and four pairs of phasiRNA-mRNA displayed negatively correlated expression changes, which may be related to bacterial wilt resistance in potato.

**Conclusions:**

This study sheds lights on the regulatory roles of small RNAs in potato resistance against bacterial wilt, and will provide a theoretical foundation for the cultivation of disease-resistant potato varieties.

**Supplementary Information:**

The online version contains supplementary material available at 10.1186/s12870-025-07768-0.

## Background

Potato (*Solanum tuberosum*) is one of the most important food crops, widely cultivated and consumed as a staple for 1.3 billion people [1]. However, potato yield and quality are severely threatened by various diseases. Among these diseases, bacterial wilt, caused by *Ralstonia solanacearum* species complex (RSSC), is a devastating global bacterial disease that affects potato production in approximately 80 countries with annual economic losses over $950 million [2]. Under favorable environmental conditions, *R. solanacearum* invades plant roots through the wounds, root tips or natural open sites of lateral root emergence, colonizes the xylem tissues, and massively multiplies, ultimately leading to the wilting symptoms and plant death [3, 4]. However, there are few efficient and environmentally friendly control methods for potato bacterial wilt [5]. Breeding of potato cultivars with disease resistance against RSSC is the most efficient approach to manage potato bacterial wilt [6].

At present, disease resources against bacterial wilt have been identified in some wild species related to *S. tuberosum*, such as *S. phureja*, *S. chacoense*, *S. commersonii*, *S. acaule*, and *S. pinnatisectum* [2, 7–11]. The resistance of *S. phureja* has been transferred to cultivated potatoes through techniques like introgression breeding and somatic hybridization [2, 12]. Using a diploid population derived from *S. phureja*, *S. chacoense*, and *S. tuberosum*, researchers have identified five major and five minor bacterial wilt resistance-related quantitative trait loci (QTLs) [13, 14]. Three SSR (Simple sequence repeat) alleles related to bacterial wilt resistance were identified from the somatic hybrids derived from protoplast fusion between *S. tuberosum* and *S. chacoense* [9]. Using *S. phureja* as a bridge and successive introgressive backcrosses to *S. tuberosum*, the resistance to bacterial wilt from *S. commersonii* has been transferred into cultivated potatoes [15, 16]. The resistance against *R. solanacearum* is associated with the restriction of bacterial invasion and multiplication particularly in the stems of potato [16]. Further fine mapping of the resistant QTLs and functional characterization of the underlying resistance genes in these wild species are required for the breeding of potato resistance against bacterial wilt.

Small RNAs are a class of 18 ~ 30 nucleotide (nt) noncoding regulatory elements involved in almost all biological processes throughout the plant life cycle [17]. Based on their distinct biogenesis pathways and mechanisms of action, canonical plant small RNAs are classified into two major categories: microRNAs (miRNAs) and small interfering RNAs (siRNAs) [18]. In plants, miRNAs are a class of endogenous small RNAs that originate from *MIR* genes. *MIR* genes are transcribed by RNA polymerase II (Pol II) to generate primary miRNA (pri-miRNAs). In the nucleus, the pri-miRNAs are processed by the DICER-LIKE 1 (DCL1) protein complex into precursor miRNAs (pre-miRNAs) characterized by stem-loop structures, which are further cleaved by DCL1 complex to yield mature miRNA duplexes [17, 18]. An expanding world of plant siRNAs has been identified, such as phased, secondary siRNAs (phasiRNAs), heterochromatic siRNAs (hc-siRNAs), and natural antisense siRNAs (nat-siRNAs) [17–20]. siRNAs are derived from different sources of long, perfectly paired double-stranded RNAs (dsRNAs) by the activity of DCL enzymes [20]. Mature miRNAs or siRNAs are loaded onto an RNA-induced silencing complex (RISC) containing ARGONAUTE (AGO) proteins and other components, which mediates posttranscriptional gene silencing (PTGS) through either mRNA degradation or translational repression, or directs transcriptional gene silencing (TGS) by targeting chromatin to induce DNA methylation that represses transcription [18, 20]. In the biogenesis of phasiRNAs, protein-coding or noncoding phasiRNA-generating (*PHAS*) precursors are targeted and cleaved by miRNA-AGO protein-containing RISC to yield phasiRNAs in a precise head-to-tail arrangement [18]. Through computational survey and experimental validation, many *PHAS* precursors and miRNA triggers have been widely identified in many plant species [21]. miRNAs and siRNAs are key regulators in plant growth, development, reproduction, biotic and abiotic stress responses [17, 18, 20, 21].

Small RNAs are big players in the interactions between plants and microbes [22–25]. To detect and defend against invading pathogens, plants have armed with a two-tiered interconnected innate immune machinery: pathogen-associated molecular patterns-triggered immunity (PTI) governed by cell surface-localized pattern recognition receptors (PRRs), and effector-triggered immunity (ETI) conferred by intracellular nucleotide-binding leucine-rich repeat receptors (NLRs) [26–28]. Some miRNAs, such as *miR1507*, *miR2109*, and members of *miR482/2118* superfamily, fine-tune plant immune responses through targeting *NLR* transcripts to trigger phasiRNAs biogenesis to suppress ETI in *Medicago truncatula* and several solanaceous species [19, 29]. Some miRNAs and siRNAs directly or indirectly modulate the expression of key regulators of PTI or ETI response, including defense-related gene expression, reactive oxygen species (ROS) accumulation, and the actions of plant hormones [23]. The roles of small RNAs in the interactions between potato and microbes have been preliminarily studied. For example, many phasiRNAs are predicted to originate from *NLR* transcripts in potato [29]. Overexpression of *Stu-miR482e* enhances the sensitivity to *Verticillium dahliae* infection probably through enhancing phasiRNAs-mediated silencing of *NLRs* [30]. Upon *Phytophthora infestans* infection, the expression of *Stu-miR160* is induced in local and systemic potato leaves. *Stu-miR160* targets *auxin response factor 10* (*StARF10*) transcripts for cleavage, thereby repressing StARF10-mediated regulation of the expression of *StGH3.6*, a mediator involved in the antagonistic cross-talk between salicylic acid (SA)-mediated defense and auxin-mediated growth pathways [31]. *Stu-miR394*, *Stu-miR6149-5p*, *Stu-miR396*, and *Stu-miR166* are down-regulated during *P. infestans* infection [32]. *Stu-miR394* negatively regulates potato late blight resistance through targeting and degrading *LEAF CURLING RESPONSIVENESS* (*StLCR*) and *Alkaline/Neutral Invertases* (*StA/N-INVE*), two positive regulators of plant immunity [33]. However, our current understanding of the roles of small RNAs during potato-*R. solanacearum* interaction remains scarce.

In the current study, we comprehensively analyzed the mRNA-seq, sRNA-seq, and degradome-seq to identify key defense pathways, regulatory small RNAs and their targets involved in potato resistance against bacterial wilt, and characterized the miRNA-triggered phasiRNA production in potato upon *R. solanacearum* infection. This study provides novel insights for further investigation on the mechanisms underlying small RNAs-mediated disease resistance against bacterial wilt in potato.

## Methods

### Plant material and growth conditions

The materials used in this study were sourced from the International Potato Center (CIP). The diploid *S. phureja* SP15-65 clone (CIP703541) [34] and the resistant diploid *S. commersonii* germplasm CM804 (CIP762476) [35] were used in this study. The detail information of these materials is presented in Table S1. Potato plantlets were propagated from nodes on Murashige-Skoog (MS) medium with sucrose (30 g/L) and kept under long-day (16 h light/8 h dark) conditions at 22°C in growth chambers. Two-week-old plantlets were transferred and cultivated in jiffy pots (Jiffy International, Norway) in a growth chamber (22°C, 60% relative humidity, 16/8 h of day/night cycle) for 3–4 weeks.

### Pathogen inoculation and disease resistance assay

The *R. solanacearum* soil drench inoculation was performed as previously described [36, 37] with some modifications. The *R. solanacearum* isolate UY031, originally isolated from potato and classified as phylotype IIB [38, 39], was used in this study. UY031 suspensions were grown overnight in BG liquid medium (10 g/L bactopeptone, 1 g/L casamino acids, 2.5 g/L glucose) at 28°C. Bacterial cells were collected by centrifugation and resuspended in sterile water to OD_600_ = 0.2. The roots of 3–4-week-old potato seedling grown in jiffy pots were injured with a 1 mL pipette tip and each seedling was inoculated by soil drenching with 50 mL UY031 suspensions. Water treatment was performed as the mock inoculation. After inoculation, the seedlings were grown in a growth chamber under the following conditions: 70% relative humidity, 16 h/8 h (day/night) photoperiod, 25°C for disease symptom scoring. Three independent experiments, with at least 12 potato seedlings per experiment, were carried out. According to the disease index ranging from ‘1’ (0–25% wilting leaves) to ‘4’ (75–100% wilting leaves), the plant wilting symptoms were recorded every two days after inoculation. The survival analysis was performed as previously described [40].

The quantification of the bacterial colonization in potato was performed as previously described [41]. Two-week-old potato seedlings in good and consistent growth conditions are transferred to square plates containing MS medium without sucrose. The plates were kept vertically in a growth chamber (25°C, 16 h light/8 h dark photoperiod, 70% relative humidity) for 3 days before *R. solanacearum* inoculation. The roots of seedlings were then wounded and inoculated with 5 µL of *R. solanacearum* suspension (OD_600_ = 0.2) at roughly 0.5–1 cm from the root tip. At 3 days post inoculation (dpi), the stem segments (2 cm above the roots) from three replicate plants were collected and weighed under sterile conditions, grounded in sterile water to extract bacteria. Bacterial titer was determined by plating a serial dilution of the *R. solanacearum* suspension onto solid BG medium plates with TTC (50 mg/L). The plates were incubated at 28°C for 2 days, and then the colonies were counted. All experiments were independently repeated for three times.

### RNA analysis

Total RNAs were extracted using TRIzol reagent (Invitrogen) from potato roots. RNA was quantified using NanoPhotometer^®^ N50 (IMPLEN GMBH), and approximately 1 µg total RNA was reverse-transcribed into cDNA using HiScript Q RT SuperMix with gDNA remover (Vazyme, R123-01). cDNAs were amplified for quantitative analysis using 2 × M5 HiPer SYBR Premix EsTaq (with Tli RNaseH) (Mei5bio, MF787-01) in a LightCycler^®^ 480 II (Roche) following the manufacturer’s instructions. The house-keeping gene *elongation factor 1α* (*StEF1α*) served as an internal reference gene to normalize the expression of individual genes. The primers used for RT-qPCR are listed in Table S2.

To analyze small RNAs, 10 µg total RNAs were run on a 15% polyacrylamide/7 M urea denaturing gel, subsequently transferred to a nylon membrane (Roche, 11417240001), and cross-linked to the membrane using UV irradiation. The membrane was then hybridized with biotin-labeled DNA probes that were complementary to target small RNAs in the hybridization solution (Beyotime, R0229) at 40°C overnight. After several washes to remove excess probes, the signals of immobilized nucleic acids on the membrane were detected by Nucleic Acid Detection Module Kit (Thermo Fisher, 89880). The relative accumulation levels of target small RNAs were normalized to the abundance of *Stu-U6*. The probes are listed in Table S2.

### mRNA sequencing and data analysis

For mRNA-seq analysis, roots of potato seedlings were sampled at 5 days post inoculation with water or UY031 by soil drenching. RNAs were extracted and quantified by agarose gel electrophoresis, NanoPhotometer^®^ N50 (IMPLEN GMBH), and Qsep1 (BiOptic). RNAs with an RNA Integrity Number (RIN) over 8.0 were used for mRNA-seq. Libraries for mRNA-seq were constructed and sequenced on the MGI DNBSEQ-T7 platform by Annoroad Gene Technology (Beijing).

mRNA sequencing data analysis was performed as reported [42]. Low-quality reads were removed and adapters were trimmed to obtain clean reads, which were mapped to the potato reference genome (*Solanum tuberosum* v8.1) [43] and the genome of CM804 (*S. commersonii* CM1.0) [35]. The expression level of each gene was calculated as the FPKM value (Fragments per kilobase of transcript per million mapped reads). Genes with |Log_2_ (Fold change) | ≥ 1 and an adjusted *P* value < 0.05 were considered as differentially expressed genes. Heatmaps of selected genes were generated using TBtools-II software [44].

### Small RNA sequencing and data analysis

Small RNA libraries construction and data analysis were performed as previously reported [45]. Total RNAs were ligated to pre-adenylated 3′ adaptors by T4 RNA ligase 2, truncated KQ (NEW ENGLAND Biolabs, M0373L), annealed with the reverse transcription primer, and then ligated to 5′ RNA adaptors using T4 RNA ligase 1 (NEW ENGLAND Biolabs, M0204L). These ligated products were reverse transcribed and amplified by PCR with GXL DNA polymerase (Takara, R050A). PCR products between 135 ~ 160-bp in length were separated by electrophoresis, gel-purified, and pair-end sequenced on an Illumina Novaseq X plus platform by Annoroad Gene Technology (Beijing).

For small RNA sequencing data analysis, low-quality reads were filtered and 3′ adapter sequences were trimmed using cutadapt2.7. Clean small RNA reads between 18- to 30-nt in length were mapped to the potato reference genome (*Solanum tuberosum* v8.1) using the Bowtie software with no mismatches. Small RNA reads that mapped to the ribosomal RNAs (rRNAs), transfer RNAs (tRNAs), small nuclear RNA (snRNAs), and small nucleolar RNA (snoRNAs) were filtered. The remaining clean reads were compared with miRNA precursors in the miRbase (https://mirbase.org/) to extract known 5p- or 3p-derived miRNAs. The sRNAminer tool was applied to predict potato novel miRNAs [46]. The frequency of the miRNAs in all libraries was expressed as reads per million (RPM). 21-nt and 24-nt *PHAS* loci were identified and characterized by PhaseTank [47] using all 21-nt and 24-nt genome-matched reads. The differentially expressed miRNAs and phasiRNAs were identified based on the following parameters: |Log_2_ (Fold change) | ≥ 0.6 and adjusted *P* value < 0.05. The targets of miRNAs and phasiRNAs were predicted using CleaveLand4 [48] and psRobot [49]. The pairs between transcripts and miRNAs/phasiRNAs with alignment penalty scores ≤ 3.5 were selected for further analysis. Gene ontology (GO) and Kyoto Encyclopedia of Genes and Genomes (KEGG) analysis were performed by clusterProfiler 4.2.2.

### Degradome sequencing and data analysis

Degradome libraries were constructed and data analysis were performed as previously described [45]. In brief, total RNAs were ligated to pre-heated 5′ RNA adaptor with a *Mme *I recognition site at its 3′ end by T4 RNA ligase 1, annealed with reverse transcription primer, and then reverse transcribed by SuperScript III reverse transcriptase (Invitrogen, 18080-085). After reverse transcription, the products were purified with VAHTSR DNA clean beads (Vazyme, N411), and then pre-amplified with GXL DNA polymerase (Takara, R050A) with 5′ biotin-labeled forward primer and unlabeled reverse primer. After PCR, the products were treated with Exonuclease I (NEW ENGLAND Biolabs, M0293L). The biotin-labeled DNA products were purified from the samples using Dynabeads MyOne Streptavidin C1 beads (Invitrogen, 65001), and then digested with *Mme *I (NEW ENGLAND Biolabs, R0637L). After digestion, the DNA products bound to the beads were ligated to the pre-annealed 3′ DNA adaptor using Quick Ligation™ Kit (NEW ENGLAND Biolabs, M2200). The ligated products were finally amplified by PCR with GXL DNA polymerase (Takara, R050A). PCR products about 140-bp in length were separated by electrophoresis, gel-purified, and sequenced on an Illumina Novaseq X plus platform by Annoroad Gene Technology (Beijing).

For degradome sequencing data analysis, low-quality reads and adapter contaminants were removed. Clean reads were mapped to the potato reference genome (*Solanum tuberosum* v8.1). The spliced targets of miRNAs and phasiRNAs were identified using the CleaveLand 4.0 pipeline. The identified cleavage sites were grouped into five different categories (0–4) as previously described [45]. The identified transcripts in category 0–2 that have at least 4 degradome tags at the cleavage site were annotated as miRNA/phasiRNA targets.

### DAB and NBT staining

The accumulation levels of hydrogen peroxide (H_2_O_2_) and superoxide were detected by 3,3′-diaminobenzidine (DAB) and nitroblue tatrazolium (NBT) staining, respectively. For DAB staining, potato roots were collected and vacuum-infiltrated in DAB solution [1 mg/mL DAB, 10 mM MES, pH 3.8, 0.2% (*v*/*v*) Tween-20] for 5 min and incubated at 25°C for 8 h. For NBT staining, the roots were immersed in a solution containing 0.5 mg/mL NBT, 10 mM Tris-HCl (pH 7.4) for 4 h in the darkness. Samples were then cleared by boiling in ethanol (90%) for 0.5 ~ 2 h before photography.

### Statistical analysis

For RT-qPCR analyses and disease assays, significant differences between samples/lines and the corresponding controls were analyzed using two-tailed Student’s *t*-test for pairwise comparisons, or one-way ANOVA analysis with Tukey’s multiple comparison test as specified in the figure legends. Samples sharing lowercase letters are not significantly different. For survival analysis, statistical analysis was performed using a Log-rank (Mantel-Cox) test, and the corresponding *P* value is shown in the graph.

## Results

### CM804 displays resistance against bacterial wilt

**Fig. 1:**
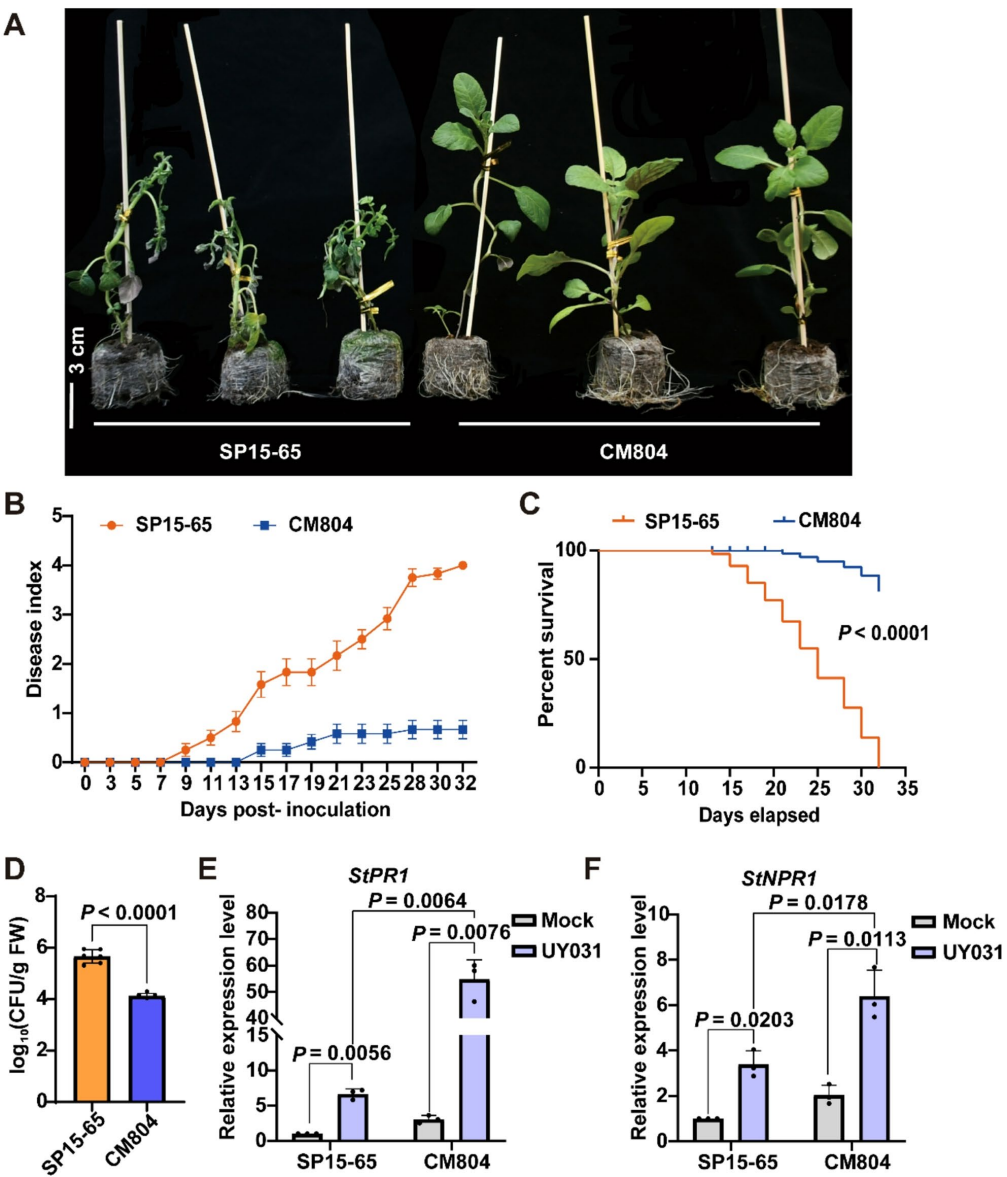
CM804 germplasm displayed resistance against *R. solanacearum* infection, while SP15-65 germplasm was susceptible to bacterial wilt. **A-C** Bacterial wilt resistance of SP15-65 and CM804 against *R. solanacearum* UY031 (OD_600_ = 0.2) in soil-drenching inoculation assays. **A **Representative images of disease symptoms of SP15-65 and CM804 plants at 25 days post inoculation. Bar, 3 cm. **B **The disease index represented the average wilting symptoms in a scale from 0 to 4. The results were shown as mean ± s.d. (*n* = 36). **C **Survival analysis of the data in (**B**). The disease indexes lower than 2 were transformed into “0”, while disease indexes ≥ 2 were transformed into “1” for each specific time point. The significant differences were analyzed by a Log-rank (Mantel-Cox) test, and the corresponding *P* value is shown in the graph. **D** Growth of *R. solanacearum* UY031 in the stems of SP15-65 and CM804. 5 µL of the bacterial solution was placed on the roots of potato seedlings (0.5–1 cm from root tip). At 3 days post inoculation, *in planta* UY031 bacterial levels were determined. **E**-**F **Levels of *StPR1 *(**E**) and *StNPR1* (**F**) were measured by RT-qPCR at 5 dpi. Data are presented as means ± s.d. (**D**-**F)**. *StEF1α* was used to normalize expression levels (**E**-**F**). Significant difference was determined by two-tailed Student’s *t* test. Exact *P* values were indicated above the bars (**D**-**F**). Experiments were independently repeated three times with similar results (**A **to** F**).

To explore and elucidate the molecular mechanisms underlying potato resistance against *R. solanacearum* infection, we assessed the resistance against bacterial wilt in diploid *S. tuberosum* group Phureja SP15-65 clone and the diploid *S. commersonii* germplasm CM804 by the soil-drenching inoculation method. Compared with CM804, stronger disease symptoms were observed in SP15-65 upon pathogen inoculation (Fig. 1A-C). To perform a more accurate assessment of bacterial wilt disease development in these two potato germplasms, we inoculated potato young seedlings with *R. solanacearum* suspensions using a root-tip-injury inoculation method on agar plates, which allows the quantification of *R. solanacearum* [41]. A significant reduction in bacterial replication was observed in the stems of CM804, compared with that of SP15-65 (Fig. 1D). The salicylic acid (SA) defense pathway is known to contribute to plant defense against bacterial pathogens [50]. We found that after the inoculation with UY031, CM804 plants displayed higher inductions of the SA signaling marker *StPR1* (*PATHOGENESIS-RELATED GENE 1*) and the SA receptor *StNPR1* (*nonexpressor of pathogenesis-related genes 1*) (Fig. 1E, F, and S1A). Collectively, these assays demonstrate that CM804 germplasm displays resistance against *R. solanacearum* infection, while SP15-65 germplasm is susceptible to bacterial wilt.

### Identification of *R. solanacearum*-responsive genes in SP15-65 and CM804 upon *R. solanacearum* invasion

To elucidate the transcriptional regulatory networks underlying potato response to *R. solanacearum* invasion on a genome-wide scale, potato germplasms SP15-65 and CM804 were inoculated with UY031 using the soil-drenching method. At 5 days post-inoculation (dpi), their transcriptomes were analyzed to examine changes in gene expression in response to *R. solanacearum* inoculation. The clean reads were mapped to the *S. tuberosum* Group Phureja DM genome (*Solanum tuberosum* v8.1), with a mapping efficiency ranging from 87.84 to 99.30% (Table S3). 3,434 differentially expressed genes (DEGs) were identified between the mock and UY031-inoculated SP15-65 plants, while 1,045 DEGs were identified in CM804 after UY031 infection (Fig. S1B and C; Table S4). We found 7,652 DEGs between UY031-inoculated SP15-65 and CM804 (Fig. S1D; Table S4). There were 152 *R. solanacearum*-responsive genes common to the three comparison groups, along with 1871 unique *R. solanacearum*-responsive genes for SP15-65, 377 unique *R. solanacearum*-responsive genes for CM804, and 6013 unique *R. solanacearum*-responsive genes between UY031-inoculated SP15-65 and CM804 (Fig. S1E). To confirm the DEGs identified from high-throughput sequencing, three DEGs were chosen at random for validation through RT-qPCR. The expression patterns of the chosen DEGs as determined by RT-qPCR were in line with those obtained through high-throughput sequencing (Fig. S2), suggesting the reliability of the high-throughput sequencing data.

The DEGs identified above were then subjected to Gene Ontology (GO) and Kyoto Encyclopedia of Genes and Genomes (KEGG) analysis. There were multiple identical significantly enriched GO terms in the three comparison groups, such as “Response to stimulus”, “Response to stress”, “Response to chemical”, and “Response to organic substance” (Fig. S3). Among these GO terms, “Response to stimulus” and “Response to stress” were the two GO terms with the largest number of genes (Fig. S3). Some DEGs between UY031-inoculated SP15-65 and CM804 were found to be specifically enriched in immunity-related GO terms, including “Response to biotic stimulus”, “Response to hormone”, and “Defense response” (Fig. S3). In addition, KEGG pathway enrichment analysis showed that the DEGs were mainly enriched in pathways such as “Metabolism (Sugar, lipid, amino acid, energy, starch, and sucrose metabolism)”, “Environmental adaption”, “Biosynthesis of other secondary metabolites”, and “Plant-pathogen interaction” (Fig. S4). Through K-means clustering analysis, the expression patterns of DEGs were grouped into 6 clusters. Among these clusters, clusters 1 and 6 contained DEGs with decreased expression in UY031-inoculated CM804 compared with UY031-inoculated SP15-65, which were enriched in pathways such as “Secondary metabolic process”, “RNA processing”, and “Transport” (Fig. S5). Clusters 2 and 4 comprised DEGs with increased expression in UY031-inoculated CM804, which were enriched in pathways such as “Primary metabolic process” and “Protein metabolic process” (Fig. S5).

To analyze the contributions of CM804-specific transcripts in potato resistance against bacterial wilt, we mapped the clean reads to the haplotype-resolved genome of CM804 [35], with an overall mapping efficiency ranging from 88.24 to 99.59% (Table S3). 2930 Hap1-mapped and 2960 Hap2-mapped DEGs were identified in SP15-65, and 952 Hap1-mapped and 954 Hap2-mapped DEGs were identified in CM804 after UY031 infection (Fig. S6A and B, Table S4). 9739 Hap1-mapped and 9670 Hap2-mapped DEGs were identified between UY031-inoculated SP15-65 and CM804 (Fig. S6A and B, Table S4). There were 125 Hap1-mapped and 113 Hap2-mapped DEGs common to the three comparison groups (Fig. S6C and D). Among the DEGs-enriched GO terms, “Response to stimulus” and “Response to stress” were still the top two GO terms (Fig. S6E and F). We further found that 1613 Hap1-mapped and 1472 Hap2-mapped transcripts were specially expressed in CM804 (Table S5), with 12 Hap1-mapped and 11 Hap2-mapped DEGs being identified between the mock and UY031-inoculated CM804 plants (Fig. S7A and B, Table S5). These CM804-specific DEGs encoded Kunitz trypsin inhibitors, PPPDE domain-containing protein, UDP-glycosyltransferase, acetyltransferase, exonuclease, FAD binding domain containing protein, carboxypeptidase A inhibitor, serine proteinase inhibitor, etc. (Table S5). *StMLP1* (*Miraculin-like protein 1*), encoding a Kunitz trypsin inhibitor, is up-regulated upon *R. solanacearum* infection, and specifically expresses in vascular bundles to enhance potato resistance [36]. The roles of these CM804-specific DEGs in plant immunity need to be further investigated.

**Fig. 2:**
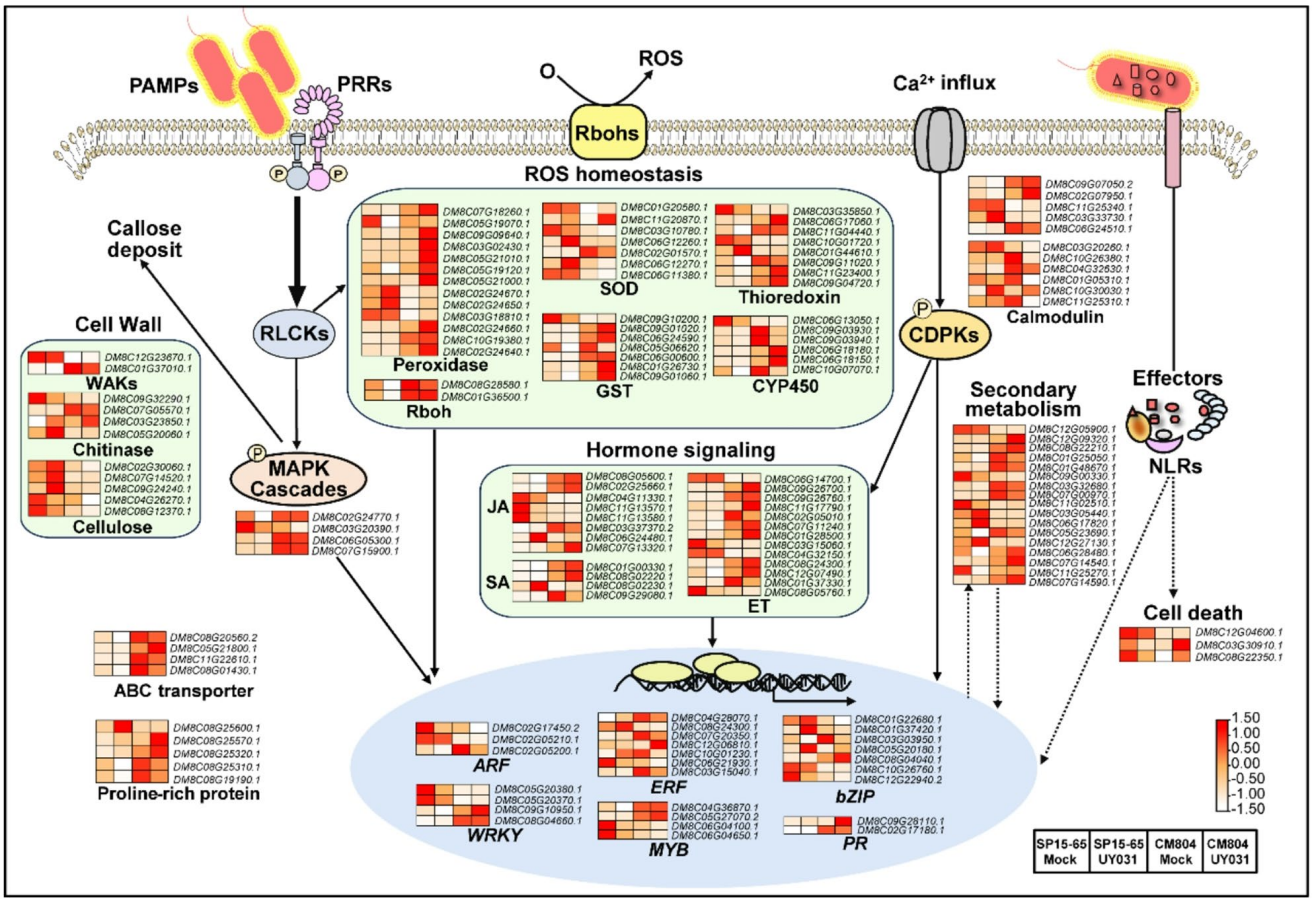
MapMan overview of the differentially expressed genes (DEGs) in SP15-65 and CM804 upon *R. solanacearum* infection, which may be involved in biotic stress responses. Red represents up-regulated expression and white represents down-regulated expression. The color scale represents the Z-score. Abbreviations are listed in Table S11.

To systematically explore the immunity-related pathways in our study, the responsive genes belonging to families known to be involved in the plant defense were scanned and selected for Mapman analysis. In the overview of plant defense pathways constructed, DEGs were enriched in diverse defense responses such as the activation of mitogen-activated protein kinase (MAPK) cascade, reactive oxygen species (ROS) homeostasis, calcium signaling, hormone signaling, secondary metabolism, and transcriptional reprogramming for defense (Fig. 2). ROS signaling plays vital roles in plant defense against vascular pathogens [51]. Consistent with the differential expression of ROS-related genes, DAB and NBT staining revealed increased accumulation of H_2_O_2_ and O_2_^•^_¯_ in UY031-inoculated CM804 (Fig. S8). Taken together, these results suggest the divergent transcriptional reprogramming in SP15-65 and CM804 modulates differential responses to *R. solanacearum* infection.

### Identification and expression profiling of **m**iRNAs and their target genes

**Fig. 3:**
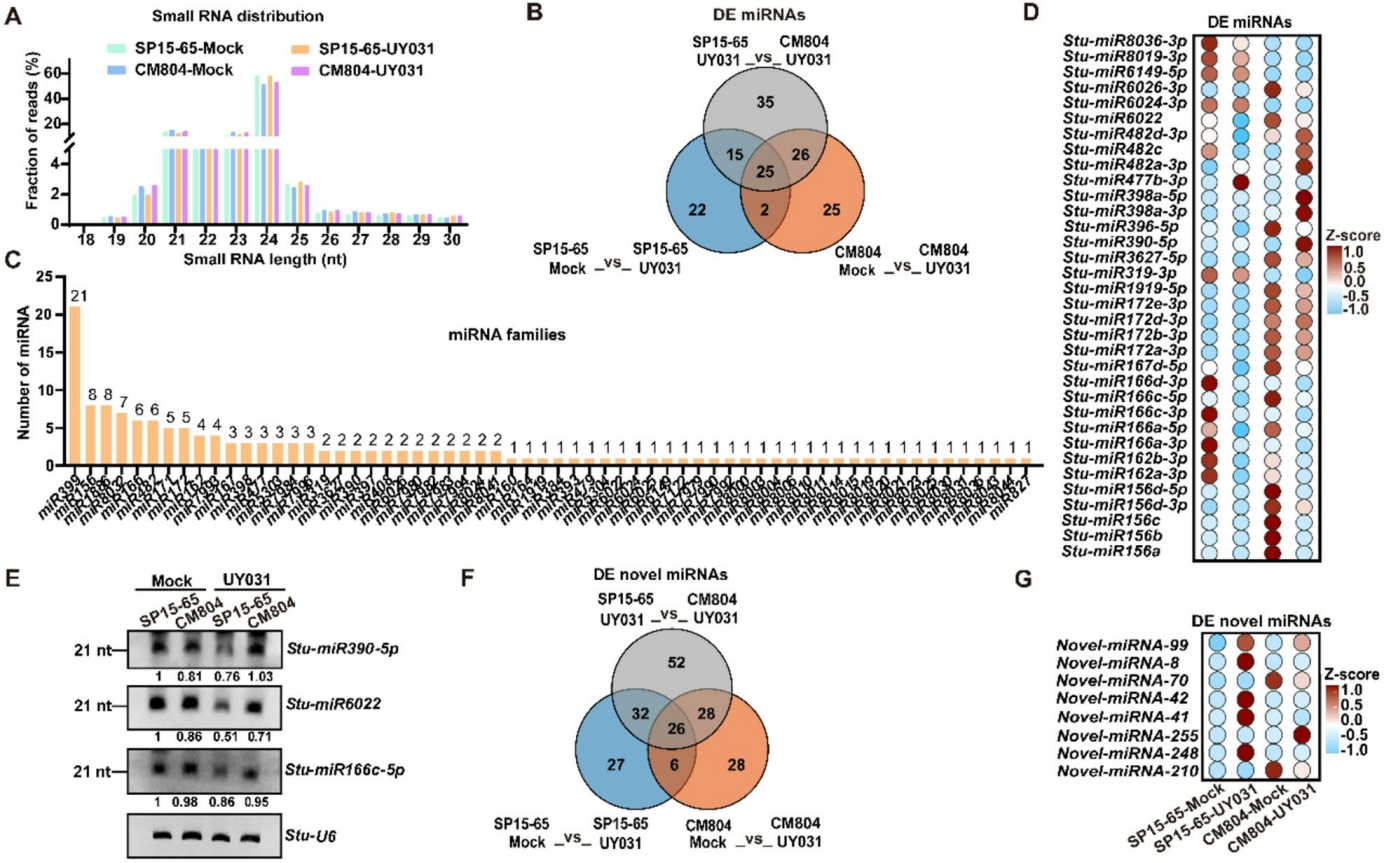
Identification of miRNAs and their differential expression analysis. **A **The length distribution of small RNAs in mock or UY031-inoculated SP15-65 and CM804. **B **Venn diagram for known miRNAs differentially accumulated in the different samples. **C **The families of differentially expressed miRNAs. **D **Expression profiles of differentially expressed known miRNAs in SP15-65 and CM804 upon pathogen inoculation. **E **Normalized abundance of *Stu-miR390-5p*, *Stu-miR6022*, and *Stu-miR166c-5p* in the indicated samples. *Stu-U6* was used as a loading control. The intensity of the blots was quantified. Experiments were independently repeated two times with similar results. Source data are provided in Table S12. **F **Venn diagram of differentially expressed novel miRNAs in SP15-65 and CM804 upon pathogen inoculation. **G **The heatmap of differentially expressed novel miRNAs in the indicated samples. Data are presented as heatmap of Z-score (**D**, **G**).

To evaluate the roles of small RNAs in the disease resistance against bacterial wilt in potato, 12 small RNA libraries of SP15-65 and CM804 germplasms under normal condition or UY031 infection were constructed and sequenced. A total of 152.71 million clean reads within 15–40 nt in length was obtained from these samples for further analysis (Table S6). The sRNA-seq analysis revealed a similar size distribution profile in SP15-65 and CM804, and the majority of small RNAs were 21- or 24-nt in length (Fig. 3A). We identified 343 known miRNAs (Table S7). 64 differentially expressed miRNAs (DEM) were identified between the mock and UY031-inoculated SP15-65 plants, while 78 DEMs were identified in CM804 after UY031 infection, with 101 DEMs between UY031-inoculated SP15-65 and CM804 (Fig. 3B, Table S7). These 150 unique DEMs were clustered into 62 families according to sequence similarity (Fig. 3C). Among these families, *miR399* was the largest family with 21 members, followed by *miR156* and *miR1886* with 8 members each (Fig. 3C). *Stu-miR156d-3p*, *Stu-miR156d-5p*, *Stu-miR156c*, *Stu-miR156b*, *Stu-miR156a*, *Stu-miR396-5p*, *Stu-miR167d-5p*, *Stu-miR6026-3p*, and *Stu-miR166c-5p* were down-regulated in CM804 upon UY031 infection (Fig. 3D). The expression levels of *Stu-miR8036-3p*, *Stu-miR8019-3p*, *Stu-miR6149-5p*, *Stu-miR6024-3p*, and *Stu-miR319-3p* were higher in SP15-65 than those in CM804 (Fig. 3D). Upon pathogen infection, the accumulation levels of *Stu-miR6022*, *Stu-miR482d-3p*, *Stu-miR482c*, *Stu-miR482a-3p*, *Stu-miR398a-5p*, *Stu-miR398a-3p*, and *Stu-miR390-5p* were higher in CM804 than those in SP15-65 (Fig. 3D). To further validate the reliability of the data obtained through sRNA-seq analysis, we next used small RNA gel blots to examine the expression patterns of *Stu-miR390-5p*, *Stu-miR6022*, and *Stu-miR166c-5p*. Consistently, the signals of *Stu-miR390-5p* and *Stu-miR6022* were stronger in UY031-inoculated CM804 than that in UY031-challenged SP15-65, while the abundance of *Stu-miR166c-5p* was slightly higher in CM804 than that in SP15-65 upon pathogen inoculation (Fig. 3E). Further, based on the method for novel miRNA prediction, a set of 321 putative unique novel miRNAs with their hairpin precursors were obtained (Table S7). A total of 147 differential expressed novel miRNAs was identified in SP15-65 or CM804 (Fig. 3F, Table S7). For example, *Novel_miRNA_8*, *Novel_miRNA_41*, *Novel_miRNA_42*, and *Novel_miRNA_248* were specifically up-regulated in UY031-challenged SP15-65 (Fig. 3G). Upon UY031 infection, the accumulation level of *Novel_miRNA_99* was increased both in SP15-65 and CM804 (Fig. 3G). *Novel_miRNA_70* and *Novel_miRNA_210* were down-regulated, while *Novel_miRNA_255* was up-regulated in UY031-inoculated CM804 (Fig. 3G).

### Identification of miRNA targets by degradome analysis

Many miRNAs modulate the expression of target genes through mRNA degradation [18, 20]. To validate the targets of miRNA in potato, we performed degradome sequencing with high throughput (Table S6). In total, 296 and 383 unique transcripts were cleaved by 100 known and 101 novel miRNAs, respectively (Table S8). The differentially expressed target genes of known and novel miRNAs were then subjected to Gene GO analysis. GO enrichment analysis revealed that these DEGs were enriched in GO terms, such as “Response to stress”, “Defense response”, “Regulation of defense response to virus”, and “Lignin catabolic process” (Fig. S9A and B). These results suggest that miRNAs-mRNA pairs may modulate plant signal transduction and metabolic processes to regulate plant defense against *R. solanacearum* infection.

**Fig. 4:**
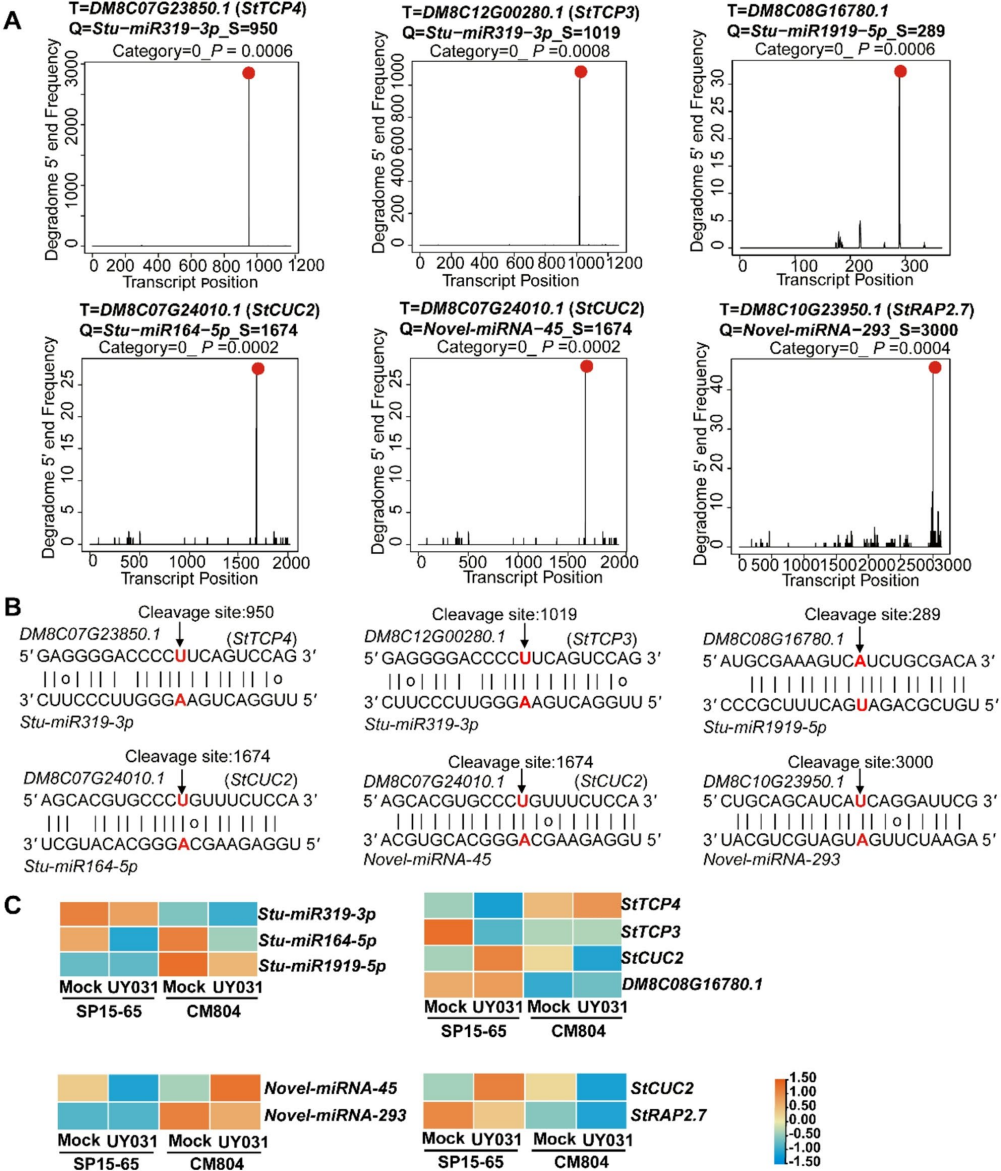
Representative miRNAs that direct target mRNA cleavage. **A **Plots showing the distribution of the degradome tags along representative miRNA targets. The nucleotide position of each target mRNA (5’−3’) is shown in the x axis. The y axis represents the relative 5’ end frequency of degradome tags. The red dots indicate miRNA-directed cleavage sites. **B **Sequence alignment of miRNAs and their respective targets that displayed negatively correlated expression changes. The cleavage sites detected in the degradome are highlighted in red letters. **C **The expression levels of representative miRNAs and target genes in SP15-65 and CM804 upon pathogen inoculation. The color scale represents the Z-score. Abbreviations for target genes are listed in Table S11.

Degradome-seq analysis revealed 6 pairs of differentially expressed miRNAs and their target genes (Category = 0; Fig. 4A and B). These pairs including *Stu-miR319-3p* and *StTCP3* (*TCP family transcription factor 3*) or *StTCP4*, *Stu-miR164-5p* and *StCUC2* (*CUP-SHAPED COTYLEDON 2*), *Stu-miR1919-5p* and *DM8C08G16780.1*, *Novel-miRNA-45* and *StCUC2*, and *Novel-miRNA-293* and *StRAP2.7* (*RELATED TO AP2.7*) (Fig. 4A and B). These 6 miRNA-mRNA pairs exhibited negatively correlated expression changes, in which the expression level of target mRNA was higher when miRNA expression was lower, and vice-versa (Fig. 4C). The expression level of *Stu-miR319-3p* in SP15-65 was higher than that of CM804 after UY031 infection, whereas its targets *StTCP3* and *StTCP4* accumulated to higher levels in CM804 (Fig. 4C). *Stu-miR164-5p*, *Stu-miR1919-5p*,*Novel-miRNA-45* and *Novel-miRNA-293* expressed at higher levels in CM804 than in SP15-65 upon pathogen inoculation, whereas their respective targets *StCUC2* [*Stu-miR164-5p* and *Novel-miRNA-45*], *DM8C08G16780.1* [*Stu-miR1919-5p*], and *StRAP2.7* [*Novel-miRNA-293*] were down-regulated in *R. solanacearum*-infected CM804 (Fig. 4C).

### Identification and annotation of *PHAS* loci and *R. solanacearum*-responsive phasiRNAs in potato

**Fig. 5:**
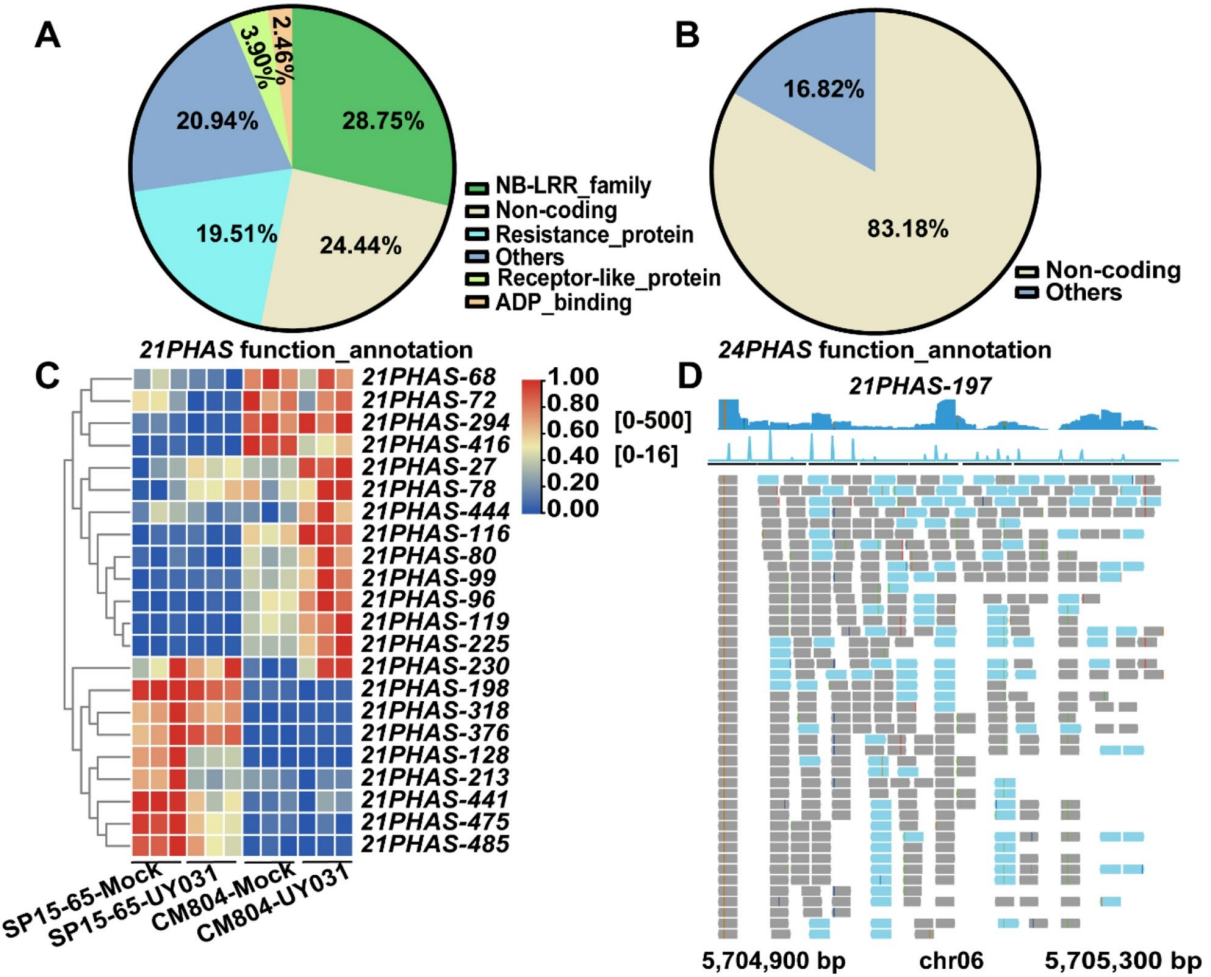
Profiling of ***PHAS*** and representative phasiRNAs in potato. **A**-**B** Pie charts showing the classification of *21PHAS* (**A**) and *24PHAS* (**B**). All *PHAS* loci were grouped into protein-coding and non-coding transcripts, and based on their annotation, coding *21PHAS* loci were further classified. **C **Heatmap showing the expression profiles of differentially expressed protein-coding *21PHAS* loci. The color scale represents the Z-score. **D **Genome browser views of phasiRNA signals at *21PHAS-197*, which were triggered by *Stu-miR482*. Blue and gray blocks represent 21-nt phasiRNAs and phasiRNAs with other lengths, respectively.

PhasiRNAs are important players in plant stress responses [18, 21]. To explore the *R. solanacearum*-responsive phasiRNAs in potato, we first performed a genome-wide identification and annotation of *PHAS* loci. A total of 487 and 761 *PHAS* were predicted to generate 21-nt and 24-nt phasiRNAs, respectively, in SP15-65 and CM804 germplasms (Table S9). About 24.44% of *PHAS* loci generating 21-nt phasiRNAs (Referred to *21PHAS*) was non-coding loci. Among the coding loci, the majority showed sequence similarity to *NB-LRR* genes (28.75%) or disease-resistance proteins (19.51%), with other proportions mainly represented by genes coding for receptor-like or ADP-binding protein (Fig. 5A). Most *24PHAS* (83.18%) were non-coding loci (Fig. 5B). In the protein-coding *21PHAS* loci, 13 *PHAS* displayed higher accumulation in CM804, while 8 *PHAS* accumulated to a higher level in SP15-65 upon pathogen inoculation (Fig. 5C), suggesting potential roles of *21PHAS* in potato resistance against bacterial wilt.

We next analyzed the miRNA triggers of phasiRNAs production from the *21PHAS* loci. *miR6024*, *miR482*, and *miR390* family members were found to trigger the biogenesis of most 21-nt phasiRNAs (Table S10). *miR482* and *miR6024* family were predicted to be involved in the cleavage of 117 and 94 *21PHAS*, respectively (Table S10). Degradome-seq analysis confirmed the significant accumulation of cleaved fragments at the cleavage sites. Notably, the phasiRNAs originating from the *Stu-miR482*-targeted *21PHAS-197* showed the highest abundance (Fig. 5D). To gain further insight, we identified the target genes of 21-nt phasiRNAs whose biogenesis were triggered by *Stu-miR482* or *Stu-miR6024* through degradome-seq analysis (Table S10), and performed GO analysis of these target genes. GO analysis showed that the targets of 21-nt phasiRNAs triggered by *Stu-miR482* or *Stu-miR6024* were enriched in GO terms, such as “Response to stimulus”, “Defense response”, and “Response to biotic stimulus” (Fig. S10A and B).

**Fig. 6:**
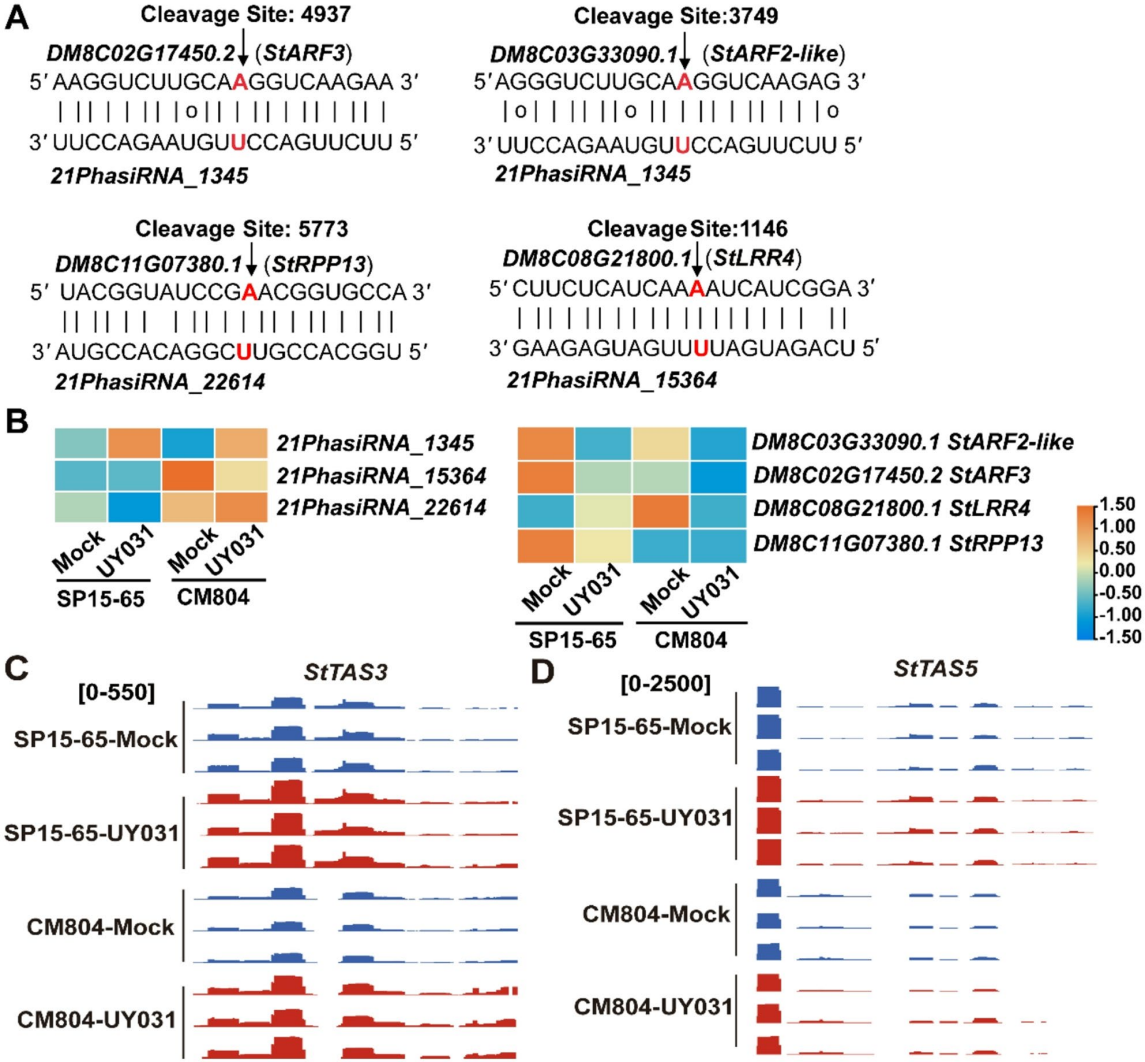
Representative PhasiRNAs that direct target mRNA cleavage. **A **Sequence alignment of PhasiRNAs and their respective targets that displayed negatively correlated expression changes. The cleavage sites detected in the degradome are highlighted in red letters. **B **The expression levels of PhasiRNAs and target genes in SP15-65 and CM804 upon pathogen inoculation. The color scale represents the Z-score. Abbreviations for target genes are listed in Table S11. **C-D** Genome browser view of *StTAS3* (**C**) and *StTAS5* (**D**)-derived tasiRNAs in SP15-65 and CM804 upon mock or UY031 inoculation.

*Stu-miR482e* is reported to negatively modulate potato resistance against *Verticillium dahliae* infection probably through phasiRNAs-mediated silencing of *NLRs* [30]. We analyzed the 21-nt phasiRNAs whose biogenesis was triggered by *Stu-miR482*, and found that the accumulation of some phasiRNAs and their target genes displayed substantial variation in SP15-65 and CM804 (Table S10). Although some *Stu-miR482*-triggered phasiRNAs targeted *NLR* genes or disease-resistance related transcripts, most of these targets expressed at low levels in SP15-65 and CM804 upon pathogen inoculation (Table S10). Integrative analysis of RNA-seq, sRNA-seq, and degradome-seq analysis revealed that the increased expression of *Stu-miR482*-triggered *21PhasiRNA_15364* and *Stu-miR6024*-triggered *21PhasiRNA_22614* in UY031-infected CM804 compared with that of UY031-infected SP15-65, were correlated to the decreased expression of their respective target genes *StLRR4* (*LEUCINE RICH REPEAT PROTEIN 4*) and *StRPP13* (*RECOGNITION OF PERONOSPORA PARASITICA 13*) (Fig. 6A and B).

*Trans*-acting small-interfering RNAs (tasiRNAs) are a class of phasiRNAs that function in *trans* to modulate the expression of target mRNAs or noncoding transcripts [21]. Two conserved *TAS* loci, *StTAS3* and *StTAS5*, have been identified to cleaved by *Stu-miR390* and *Stu-miR482c*/*miR482a-3p*, respectively, to initiate the biogenesis of tasiRNAs in potato [52]. *Stu-miR390-5p* and *Stu-miR482c* were up-regulated in CM804 after UY031 infection (Fig. 3D). The abundance of *StTAS3*-derived tasiRNAs, but not *StTAS5*-derived tasiRNAs, significantly increased in UY031-inoculated CM804 (Fig. 6C and D). Moreover, *StTAS3*- and *StTAS5*-derived tasiRNAs accumulated to a higher level in SP15-65 than those in CM804 after UY031 invasion (Fig. 6C and D). Degradome-seq analysis revealed that negatively correlated expression changes existed in *21PhasiRNA_1345* derived from *StTAS3* and its target genes, including *StARF3* (*AUXIN RESPONSE TRANSCRIPTION FACTOR 3*) and *StARF2-like* (Fig. 6A and B, Table S10). The expression levels of *StARF3* and *StARF2-like* decreased in CM804 and SP15-65 after pathogen infection, along with increased levels of *21PhasiRNA_1345* (Fig. 6A and B). Some tasiRNAs derived from *StTAS3* and *StTAS5* were not expressed in CM804 (Table S10), which may lead to the de-repressed expression of their target genes. Despite the differential expression of some tasiRNAs upon UY031 infection, their targets did not display negatively correlated expression changes (Table S10), indicating that besides tasiRNAs, a complicated mechanism was involved in the regulation of the expression of these targets.

## Discussion

### Transcriptome response to *R. solanacearum* in the roots of *S. commersonii*

Bacterial wilt poses a huge threat to global potato production. For stable and sustainable potato production, breeding potato cultivars with bacterial wilt resistance is an efficient and economical solution. *S. commersonii* is one of the wild species carrying resistant gene resources against bacterial wilt. The molecular mechanisms underlying defense against *R. solanacearum* in *S. commersonii* remain largely unknown. In the pathogen-challenged roots of *S. commersonii* accessions resistant (F118) and susceptible (F97) to the pathogen, ethylene (ET), Jasmonic acid (JA), and SA-related genes were previously reported to be differentially expressed [53]. Consistently, we found that many DEGs participated in JA, SA, and ET signaling (Fig. 2), suggesting important roles of these defense-related hormones in plant resistance against bacterial wilt. Our study also revealed that MAPK cascade is involved in the differential responses in SP15-65 and CM804 upon UY031 infection (Fig. 2). The roles of MAPK cascade in potato immunity against *R. solanacearum* infection have been recently reported. Silencing of *StMKK1*, a component of the MAPK cascade, enhances potato defense against *R. solanacearum*, possibly through activating PTI and SA-associated immune responses [54]. However, transgenic potato seedlings overexpressing *StMAPK3* display enhanced resistance to *R. solanacearum* [37], suggesting the complex roles of MAPK cascade in plant resistance against bacterial wilt. The expression of *StMAPK3* is inhibited by a type one protein phosphatase StTOPP6, which is a negative modulator of potato resistance to bacterial wilt [37]. How hormone signaling and MAPK cascade modulate the resistance against *R. solanacearum* in CM804 remains to be investigated.

As a vital regulator of plant immunity, ROS homeostasis was differently modulated in SP15-65 and CM804 (Fig. 2), which may contribute to the accumulation of H_2_O_2_ and O_2_^•^_¯_in UY031-inoculated CM804 (Fig. S8). Some reported regulators may modulate plant immunity against bacterial wilt through maintaining ROS homeostasis and cell death. For example, tomato NADPH oxidase SlWfi1, a respiratory burst oxidase homologue (RBOHs) required for ROS production, interacts with the effector protein RipBJ of *R. solanacearum*, and promotes tomato tolerance to *R. solanacearum* [55]. The *Multiprotein bridging factor 1c* (*StMBF1c*) gene is induced by *R. solanacearum* invasion. Overexpression of *StMBF1c* in *Arabidopsis thaliana* enhanced resistance to bacterial wilt by mediating ROS accumulation and cell death [56]. The potato transcription factor gene *StNACb4* is upregulated upon *R. solanacearum* induction. Silencing its homolog *NbNACb4* in tobacco (*Nicotiana tabacum*) increased susceptibility to the pathogen, while overexpressing *StNACb4* significantly enhanced tolerance through inducing cell death and callose deposition [57]. The mechanisms underlying ROS signaling in the defense against bacterial wilt in CM804 remain obscure. ROS may not only directly repress the colonization of vascular pathogens due to its toxicity at high concentration, but also participate in the cell wall lignin biosynthesis and polymerization, vascular cell redifferentiation, and the microbiota homeostasis [51]. Further histological, genetic, and biochemical studies are needed to elucidate the vital roles of ROS signaling enabled the vascular resistance against *R. solanacearum* in potato roots.

### MiRNA/phasiRNA-mRNA regulatory network in potato upon *R. solanacearum* infection

**Fig. 7:**
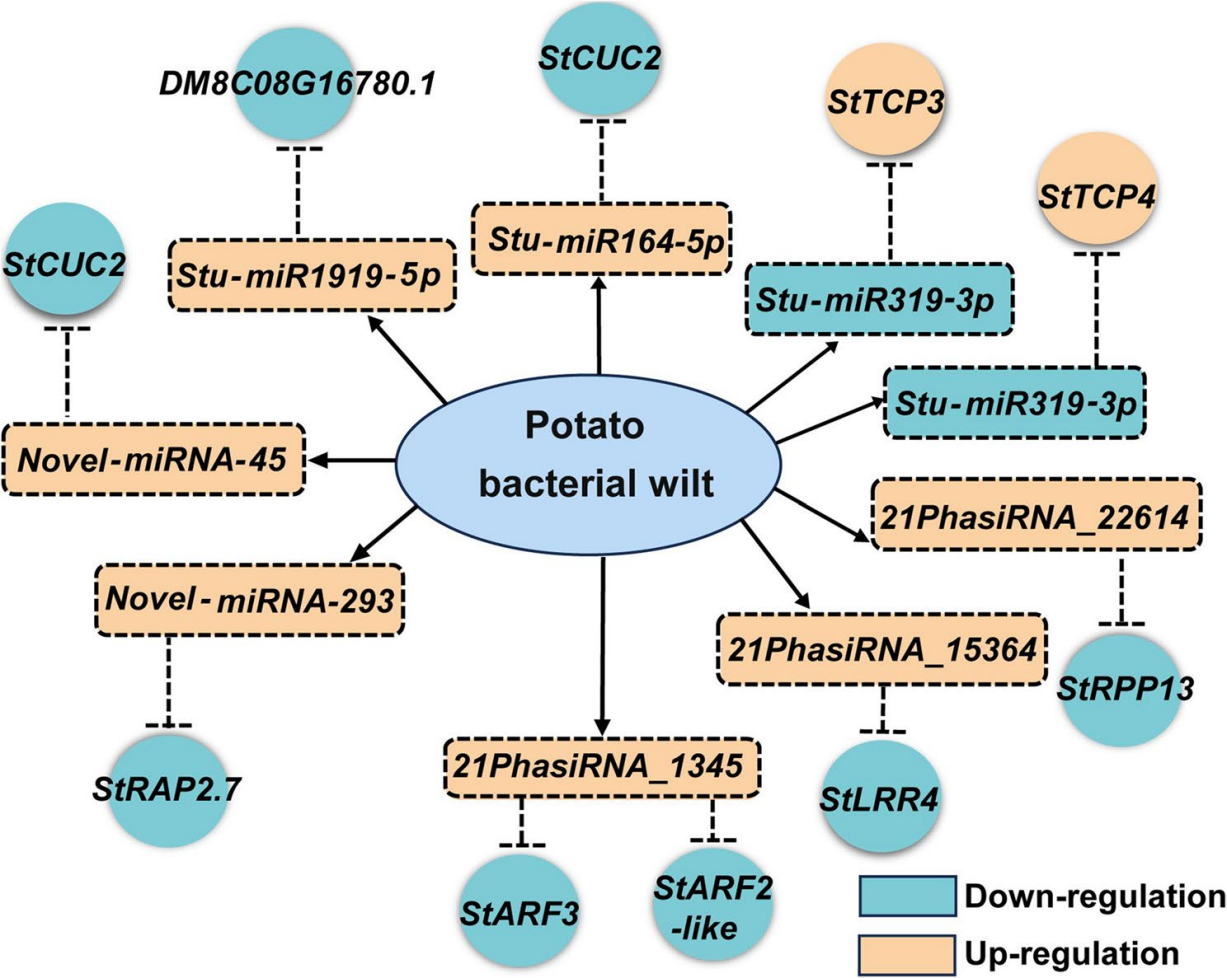
Model showing the miRNAs/phasiRNAs-mRNAs regulatory networks in response to *R. solanacearum* infection in potato miRNAs and phasiRNAs are represented in rectangular boxes, and target transcripts are represented in circles. Aqua green background represents down-regulation, while light orange background represents up-regulation. Dashed arrows represent putative negative regulatory relationships. Abbreviations for target genes are listed in Table S11

miRNAs are important components of plant immune system as they target key modulators of pathogen perception, signal transduction, and various downstream defense signaling [22–24]. Mature miRNAs often modulate the expression of target genes through either mRNA degradation or translational repression. In this study, a model of the responses to *R. solanacearum* infection based on the miRNA/phasiRNAs-mRNA regulatory network shows the potential roles of the *R. solanacearum*-responsive miRNAs and phasiRNAs in plant immunity (Fig. 7). 6 miRNA-mRNA pairs display negatively correlated expression changes (Fig. 4). Among these pairs, the known miRNAs, including *miR164*, *miR319*, and *miR1919*, have been reported to finetune plant immunity responses. In wheat (*Triticum aestivum*), *TaNAC21/22*, a target gene of *Tae-miR164*, negatively regulates wheat resistance against stripe rust [58]. However, *Arabidopsis mir164c* mutant seedlings and transgenic plants overexpressing its target *AtNAC4* display enhanced cell death symptoms in response to avirulent pathogens [59]. The fungal pathogen *Magnaporthe oryzae* (*M. oryzae*) infection induces the expression of *Osa-miR319* and suppresses its target gene *OsTCP21* expression to suppress rice immune responses, possibly through suppressing JA signaling [60]. *miR1919* negatively modulates the resistance against virus infection in *Nicotiana benthamiana* [61]. The roles of miRNA/mRNA pairs, including *Stu-miR319-3p*/*StTCP3/4*, *Stu-miR164-5p*/*StCUC2*, *Stu-miR1919-5p*/*DM8C08G16780.1*, *Novel-miRNA-45*/*StCUC2*, and *Novel-miRNA-293*/*StRAP2.7*, in potato resistance against bacterial wilt remain to be investigated.

Regulatory cascades mediated by phasiRNAs are important components in plant immunity [21–23]. In the absence of pathogen infection, *NLR*-derived phasiRNAs are proposed to finetune the expression of *NLR* transcripts to minimize the auto-activation and programmed cell death [21]. In this study, we have predicted some *NLR* genes as being able to generate siRNAs in the roots of potato (Table S10). However, most of these *NLR* genes expressed at low levels in SP15-65 and CM804 upon pathogen inoculation (Table S10), suggesting specific and complicated mechanisms that govern the expression of *NLR* genes in potato roots. The *miR390-TAS3* pathway is well known as an evolutionarily conserved regulatory circuit in land plants, which plays important roles in plant immunity [21, 42]. Our study revealed that tasiRNAs derived from *StTAS3* may participate in potato root immunity through finetuning the expression of target genes, such as *StARF3* and *StARF2-like* (Fig. 7). OsARF3s positively modulate rice disease resistance to *M. oryzae* and the bacterial pathogen *Xanthomonas oryzae* pv. *oryzae* (*Xoo*) [42]. The roles of *StARF3* and *StARF2-like* in potato responses to *R. solanacearum* infection need to be further analyzed. Although other differentially expressed miRNAs/phasiRNAs and their targets do not exhibit negatively correlated expression changes, we cannot exclude the possibility that these miRNAs/phasiRNAs modulate the expression of target genes through translational repression or interaction with proteins or other non-coding RNAs. Further ribosome profiling, epigenetic and genetic analysis will shed new light on the vital roles of the repertories of miRNAs and phasiRNAs and their putative targets in potato defense responses.

## Conclusions

Bacterial wilt is one of the most serious diseases affecting the global yield and quality of potato. This study is the first attempt to combine miRNA/phasiRNAs and mRNA expression data along with degradome-seq analysis to identify key regulatory miRNA/phasiRNAs-mRNA circuits in potato response to *R. solanacearum* infection. We have identified 115 unique known and 147 putative novel miRNAs to be differentially expressed in the susceptible germplasm SP15-65 or the resistant *Solanum commersonii* germplasm CM804. Six pairs of miRNAs and target genes, as well as four pairs of phasiRNA-mRNA display negatively correlated expression changes, which may be related to bacterial wilt resistance in potato. Overall, the comprehensive integrated analysis in this study not only delivers new insights into the transcriptome dynamics and regulatory network components of immune responses in potato roots, but also provides several characterized candidate miRNAs/phasiRNAs and their putative targets for breeding potato varieties with bacterial wilt resistance.

## Supplementary Information

Supplementary Material 1.

Supplementary Material 2. 

Supplementary Material 3.

Supplementary Material 4.

Supplementary Material 5.

Supplementary Material 6.

Supplementary Material 7.

Supplementary Material 8. 

Supplementary Material 9.

Supplementary Material 10.

Supplementary Material 11.

Supplementary Material 12.

Supplementary Material 13.

## Data Availability

All data generated or analyzed during this study are included in this published article and its supplementary information files. The mRNA, small RNA, and degradome sequencing datasets generated in this study have been deposited in the China National GeneBank DataBase (CNGBdb) [62] under project CNP0007406 (https://db.cngb.org/data_resources/project/CNP0007406/). Uncropped versions of all gels or blots are shown in Table S12.
