# Supplementary material for: Integrated analysis of transcriptome, sRNAome and degradome sequencing provides insights into bacterial wilt resistance in potato
Source: BMC Plant Biol. 2025 Dec 5;26:52. doi: 10.1186/s12870-025-07768-0 (PMC12797843; doi:10.1186/s12870-025-07768-0)
Supplement: Supplementary file 1 — Supplementary Material 1. [file 12870_2025_7768_MOESM1_ESM.docx]

**Supplementary information for**

**Integrated analysis of transcriptome, sRNAome and degradome sequencing provides insights into bacterial wilt resistance in potato**

**Yu Yang^1^**^†^**, Xiaoyuan Zhang^1^**^†^**, Jiaping Ma^1^**^†^**, Jun Xiao^1^, Zhengxiang Feng^1^, Junmei Yu^1^, Wanjie Li^1^, Pengfei Jiang^2^,** **Guangtao Zhu^3^, Yupeng Geng^4*^, Junzhong Liu^1*^**

^1^Yunnan Key Laboratory of Cell Metabolism and Diseases, Center for Life Science and School of Life Sciences, Yunnan University, Kunming 650500, China

^2^School of Life Sciences, The National Engineering Laboratory of Crop Resistance Breeding, Anhui Agricultural University, Hefei 230036, China

^3^School of Life Sciences, Yunnan Key Laboratory of Potato Biology, Yunnan Normal University, Southwest United Graduate School, Kunming 650500, China.

^4^State Key Laboratory for Vegetation Structure, Function and Construction (VegLab), Ministry of Education Key Laboratory for Transboundary Ecosecurity of Southwest China, School of Ecology and Environmental Science, Yunnan University, Kunming 650500, China

^†^Yu Yang, Xiaoyuan Zhang, and Jiaping Ma have contributed equally to this work.

**^*^**Correspondence: Yupeng Geng ([ypgeng@ynu.edu.cn](mailto:ypgeng@ynu.edu.cn)); Junzhong Liu ([liujunzhong@ynu.edu.cn](mailto:liujunzhong@ynu.edu.cn))

**Supplementary Figures S1-10**


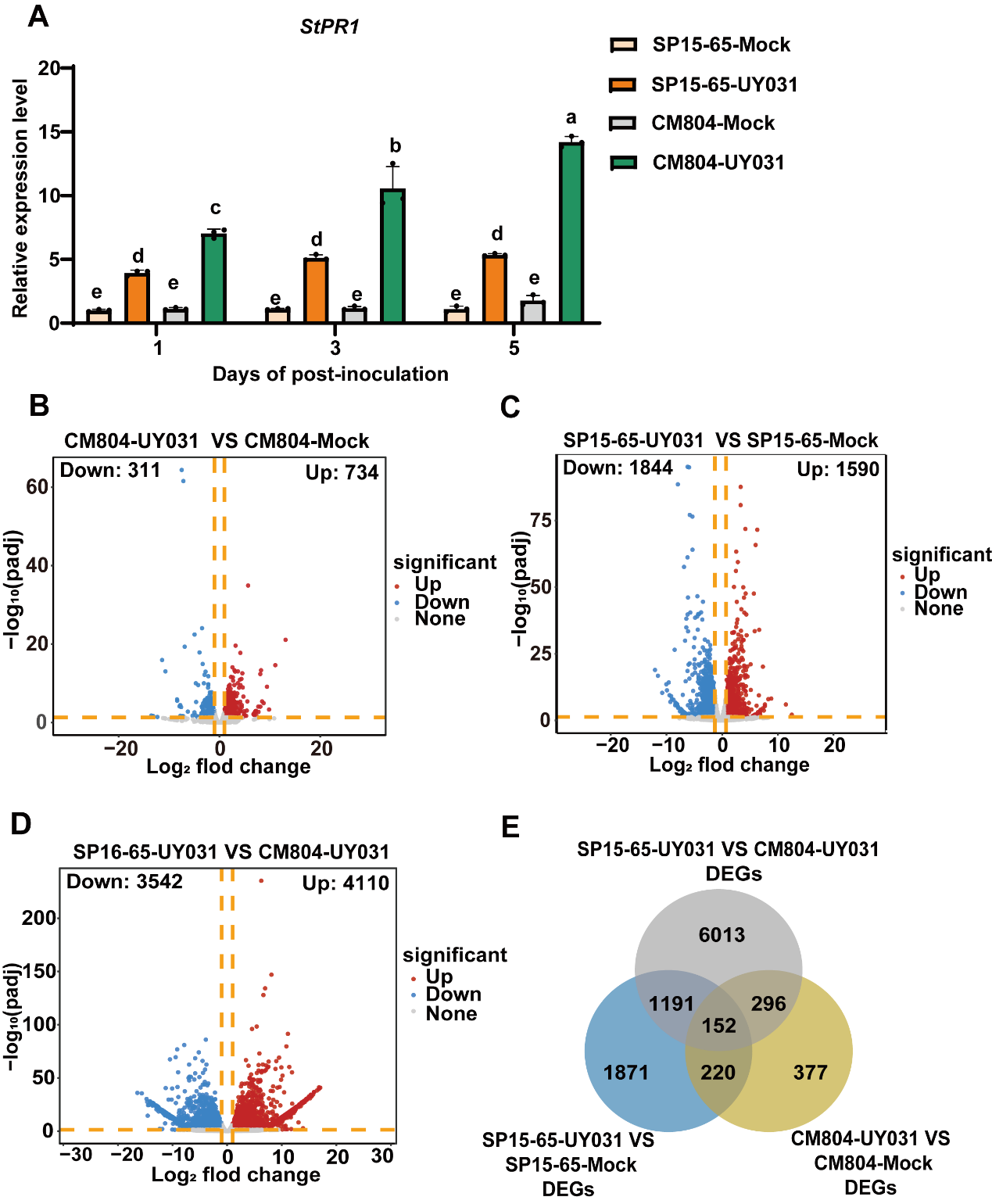


**Supplementary Figure S1.** **Levels of *StPR1*** **and the identification of DEGs in SP15-65 and CM804 upon mock or UY031 inoculation.**

(**A**) Levels of *StPR1* were measured by RT-qPCR at 1, 3, and 5 dpi. Data are presented as means ± s.d.. *StEF1α* was used to normalize expression levels. Significant difference was determined by two-way ANOVA with Tukey’s HSD post hoc analysis. Different lowercase letters indicate significant differences.

(**B**) Volcano plot of DEGs between the control group and CM804 inoculated with UY031.

(**C**) Volcano plot of DEGs between the control group and SP15-65 inoculated with UY031.

(**D**) Volcano plot of DEGs between UY031-inoculated SP15-65 and CM804.The red dots indicate significantly up-regulated genes, while the blue dots indicate significantly down-regulated genes (**B-D**).

(**E**) Venn diagram analysis of DEGs between CM804 and SP15-65 inoculated with water or UY031.


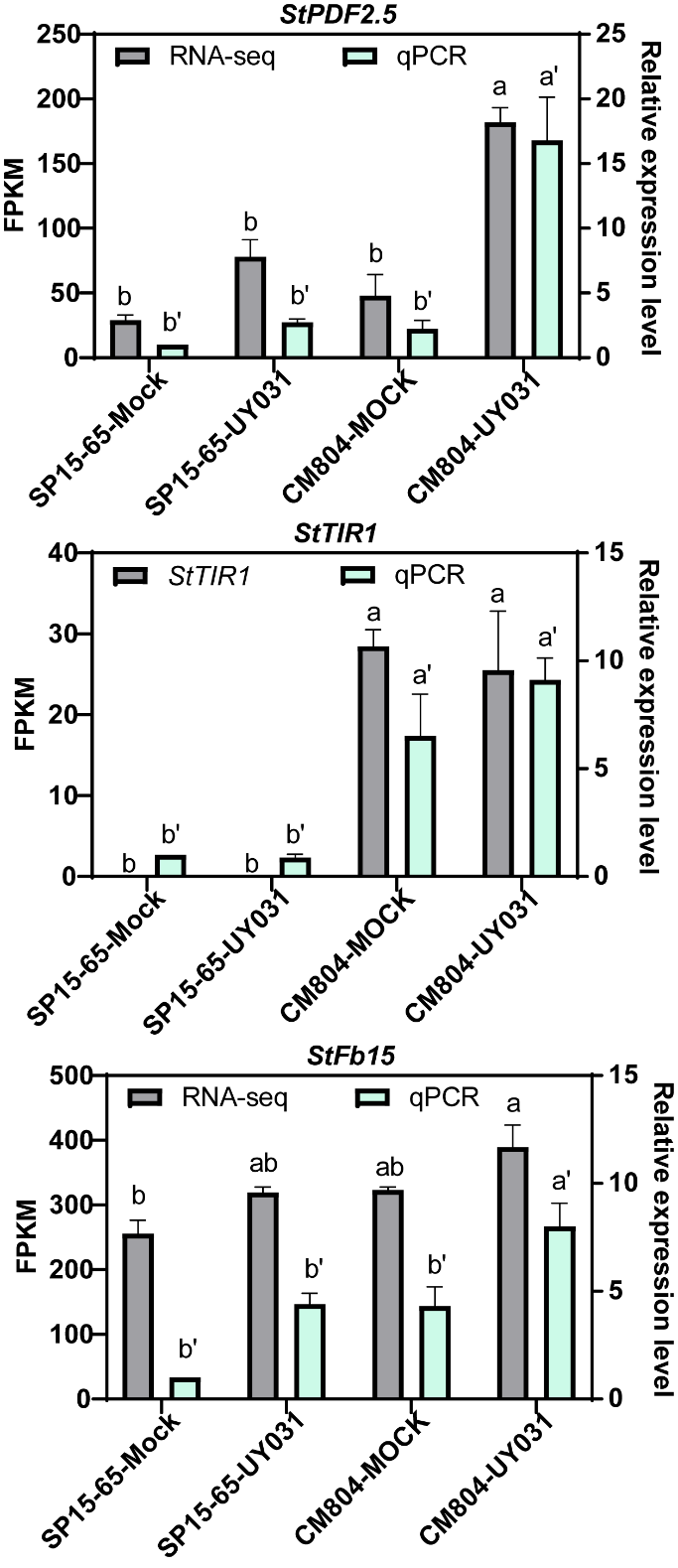


**Supplementary Figure S2. RT-qPCR validation of 3 DEGs.**

The light green and gray bars show the relative expression levels of mRNA based on RT-qPCR and high-throughput sequencing results, respectively. FPKM represents fragments per kilobase of transcript per million mapped reads. Data are presented as means ± s.d.. *StEF1α* was used to normalize expression levels. The statistically significant difference was evaluated by one-way ANOVA. Abbreviations are listed in Table S11. Experiments were independently repeated two times with similar results.


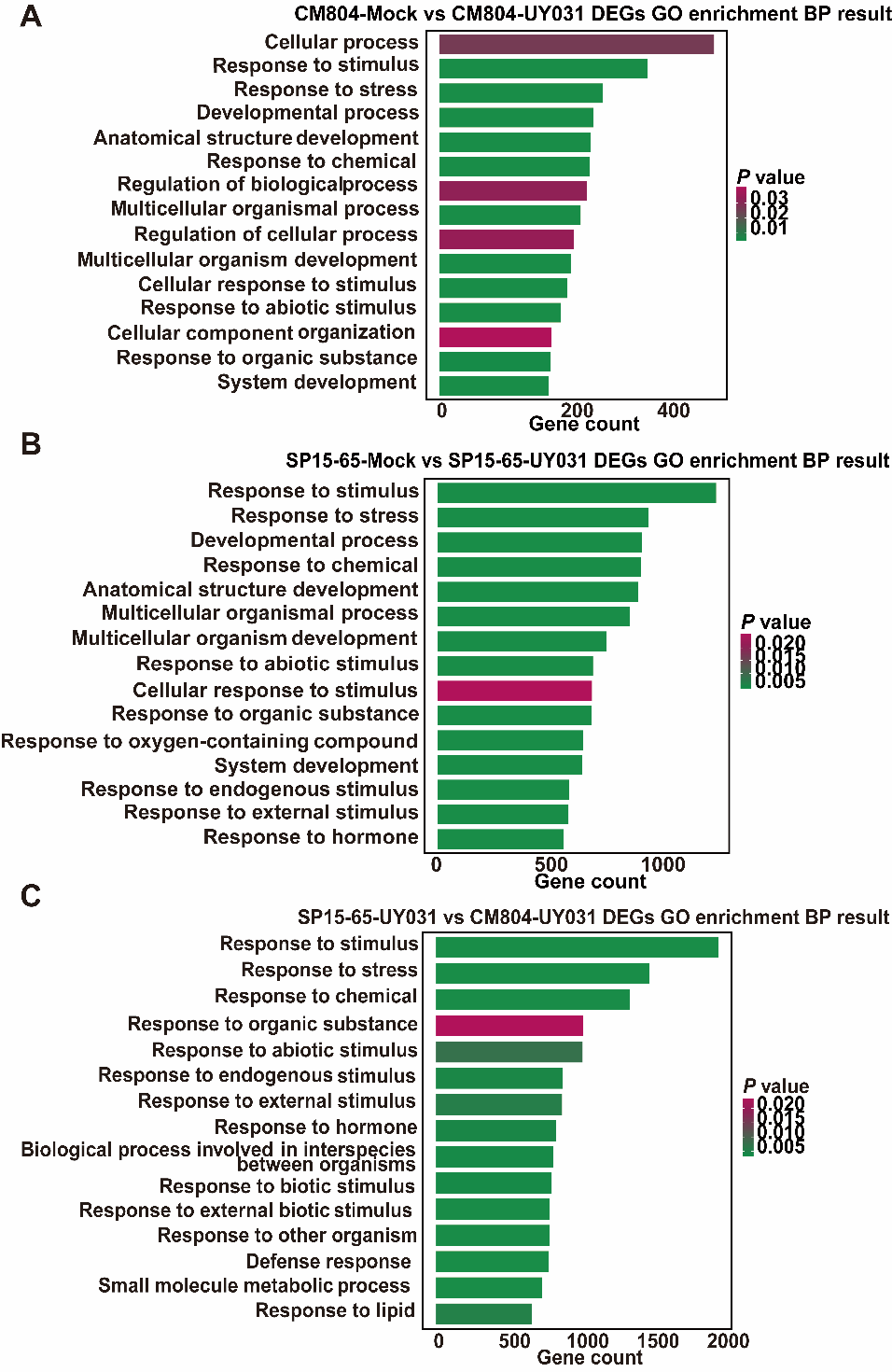


**Supplementary Figure S3. GO enrichment analysis of DEGs between CM804 and SP15-65 upon mock or UY031 inoculation.**

(**A**) GO enrichment analysis of DEGs between the control group and CM804 inoculated with UY031.

(**B**) GO enrichment analysis of DEGs between the control group and SP15-65 inoculated with UY031.

(**C**) GO enrichment analysis of DEGs between UY031-inoculated SP15-65 and CM804.

The horizontal axis represents the count of enriched genes, and the vertical axis represents the top 15 enriched GO terms (**A-C**).


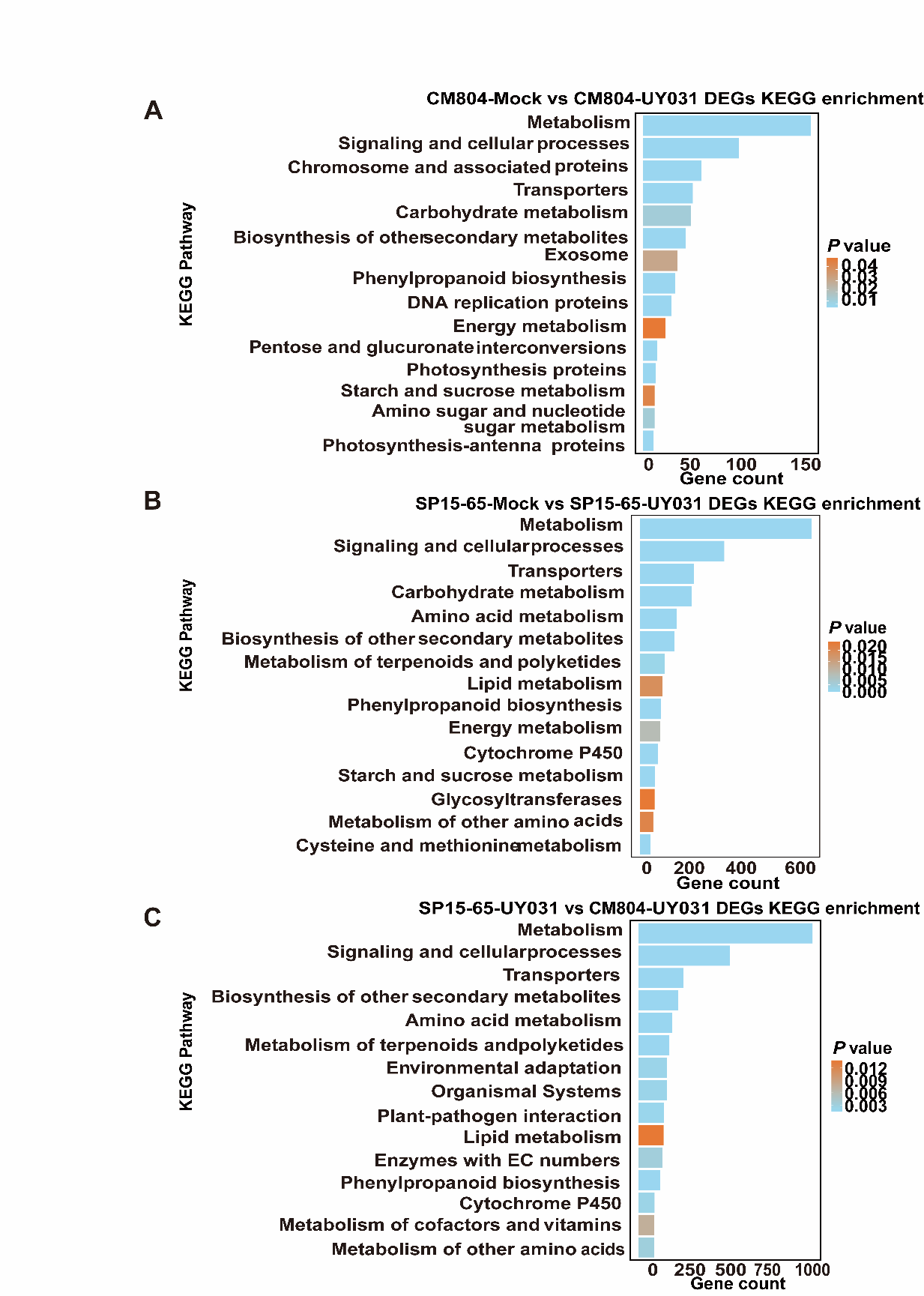


**Supplementary Figure S4. KEGG pathway enrichment analysis of DEGs between CM804 and SP15-65 upon mock or UY031 inoculation.**

(**A**) KEGG pathway enrichment analysis of DEGs between the control group and CM804 inoculated with UY031.

(**B**) KEGG pathway enrichment analysis of DEGs between the control group and SP15-65 inoculated with UY031.

(**C**) KEGG pathway enrichment analysis of DEGs between UY031-inoculated SP15-65 and CM804. The horizontal axis represents the count of enriched genes, and the vertical axis represents the top 15 enriched KEGG pathways (**A-C**).


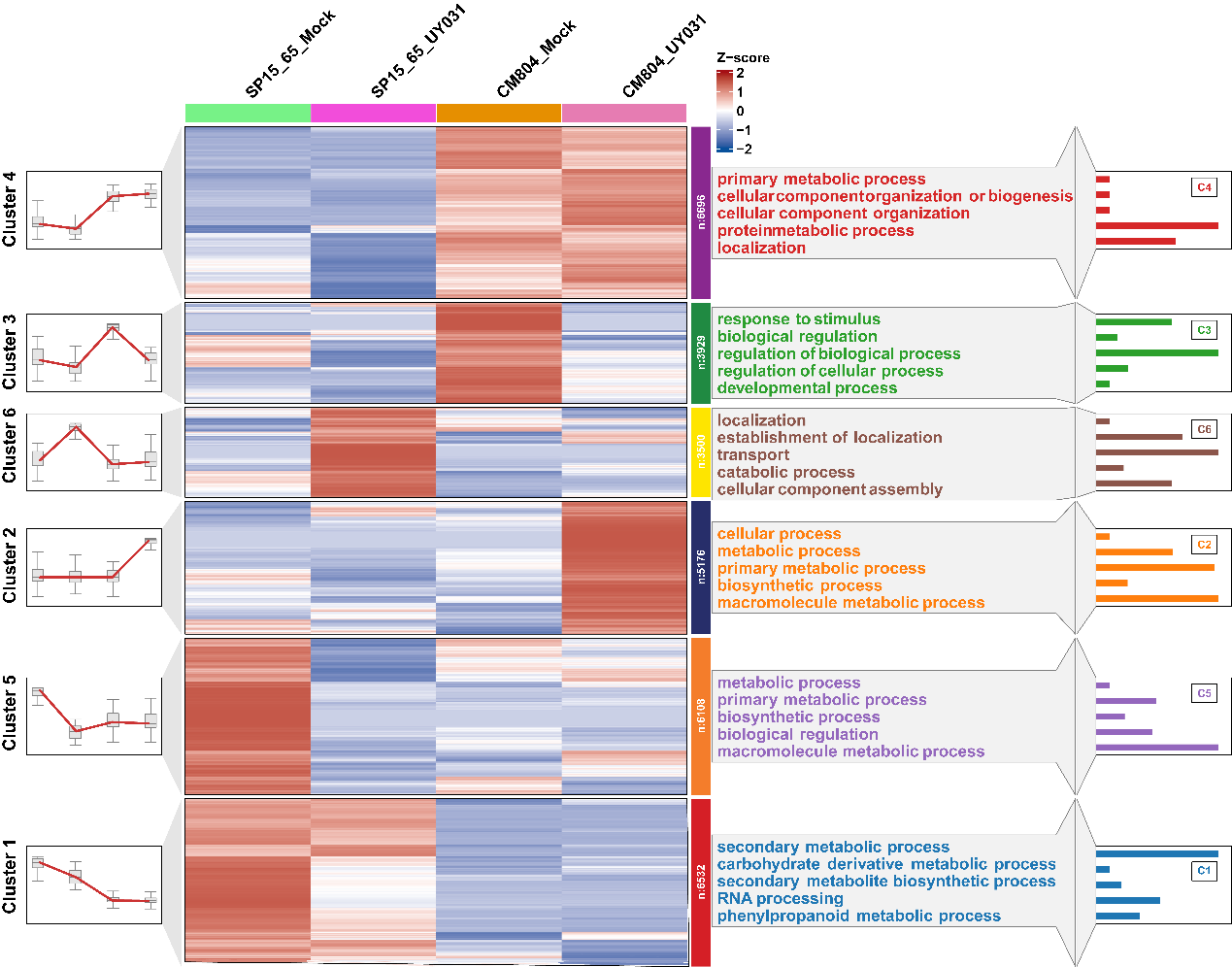


**Supplementary Figure S5. K-means clustering analysis of DEGs between CM804 and SP15-65 inoculated with water or UY031.**

Through K-means clustering analysis, the expression patterns of DEGs were grouped into 6 clusters according to their expression pattern. From left to right, the data represent the average expression pattern of each cluster, heatmaps showing the expression profiles, GO enrichment analysis, and the corresponding *P* values of each GO terms.


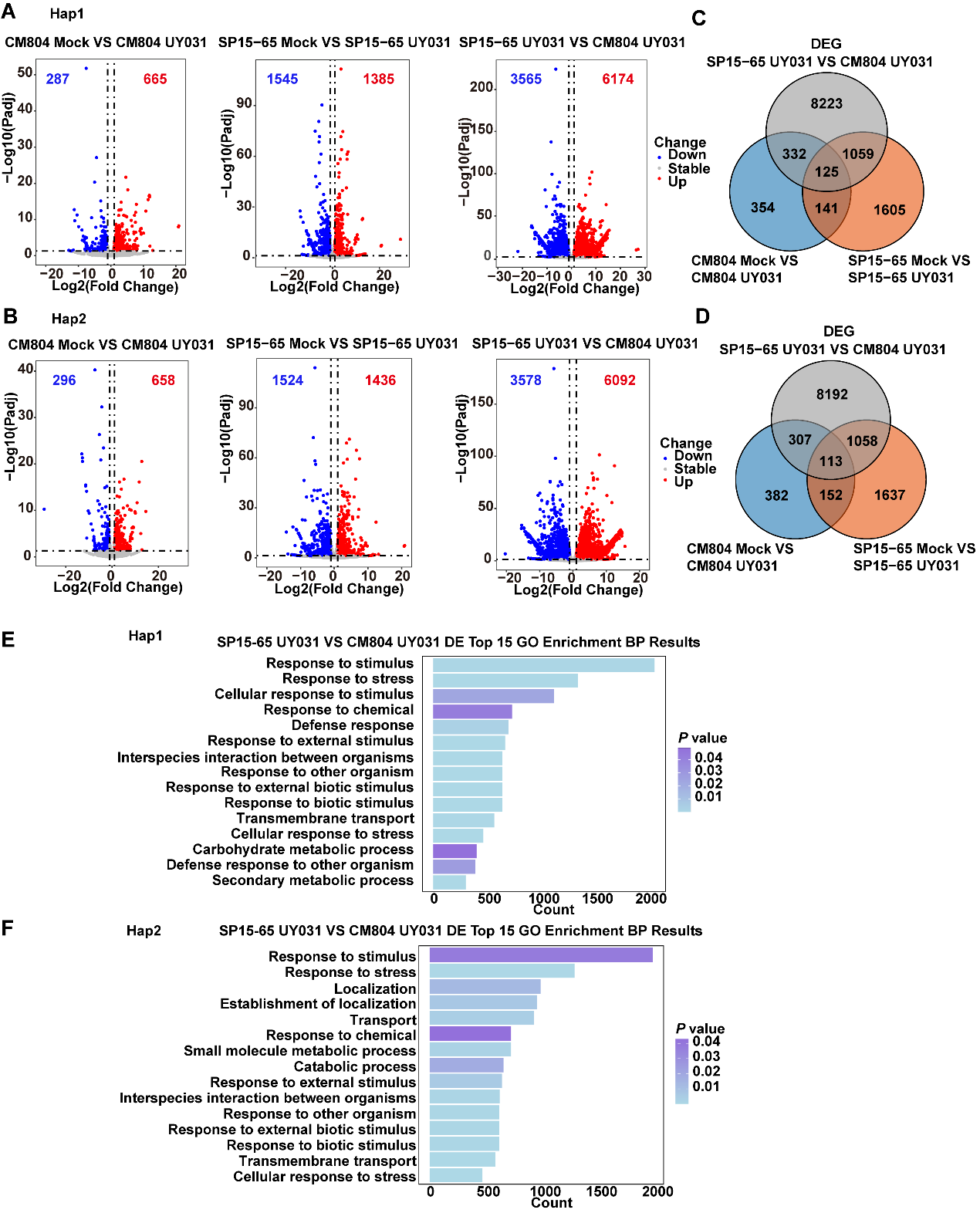


**Supplementary Figure S6. Identification of DEGs mapped to the two haplotypes of CM804 in SP15-65 and CM804 upon mock or UY031 inoculation.**

(**A-B**) Volcano plot of DEGs mapped to haplotype 1 (**A**) and 2 (**B**) of CM804 in SP15-65 and CM804 upon mock or UY031 inoculation. The red dots indicate significantly up-regulated genes, while the blue dots indicate significantly down-regulated genes (**A**-**B**).

(**C-D**) Venn diagram analysis of DEGs mapped to haplotype 1 (**C**) and 2 (**D**) of CM804 in SP15-65 and CM804 upon mock or UY031 inoculation.

(**E-F**) GO enrichment analysis of Hap1-mapped (**E**) and Hap2-mapped (**F**) DEGs. The horizontal axis represents the count of enriched genes, and the vertical axis represents the top 15 enriched GO terms (**E-F**).


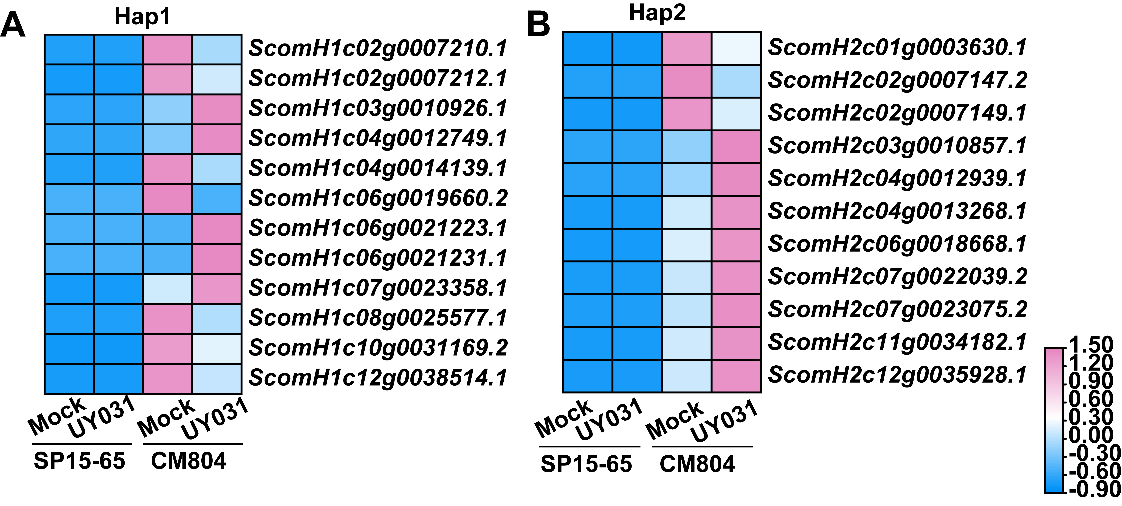


**Supplementary Figure S7. Expression pattern analysis of Hap1-mapped and Hap2-mapped DEGs that were specially expressed in CM804.**

(**A-B**) Heatmap showing the expression profiles of Hap1-mapped (**A**) and Hap2-mapped (**B**) DEGs that were specially expressed in CM804. Data are presented as heatmaps of Z-score.


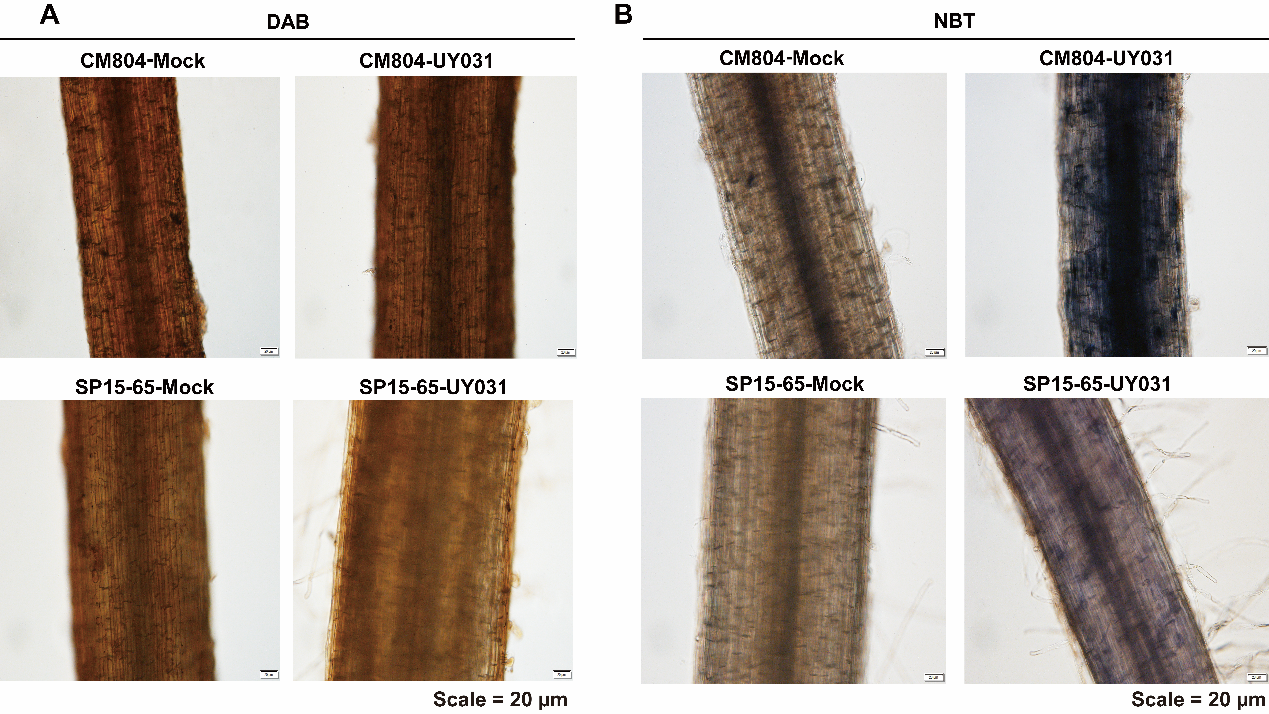


**Supplementary Figure S8. DAB and NBT staining revealed increased accumulation of H_2_O_2_ and O_2_^•^_¯_** **in UY031-inoculated CM804.**

The indicated samples were inoculated with water or UY031 (OD_600_ = 0.1) for 24 hours before DAB (**A**) or NBT (**B**) staining. Experiments were repeated two times with similar results. Scale bars, 20 μm (**A-B**).


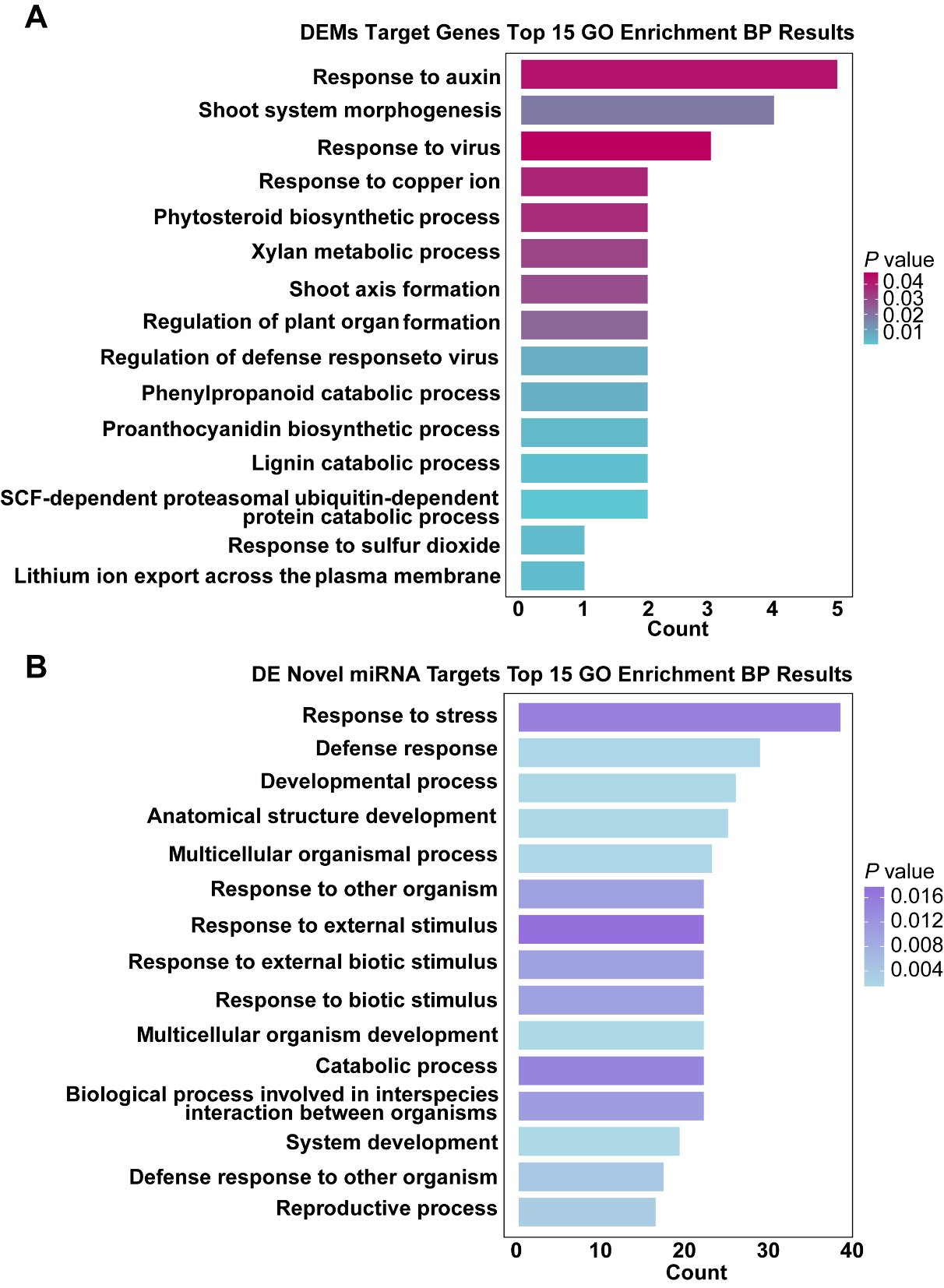


**Supplementary Figure S9. GO enrichment analysis of genes targeted by known and novel miRNAs in CM804 and SP15-65 inoculated with water or UY031.**

(**A**) GO enrichment analysis of genes targeted by known miRNAs in CM804 and SP15-65 inoculated with water or UY031.

(**B**) GO enrichment analysis of target genes of novel miRNAs in CM804 and SP15-65 inoculated with water or UY031. The horizontal axis represents the count of enriched genes, and the vertical axis represents the top 15 enriched GO terms (**A-B**).


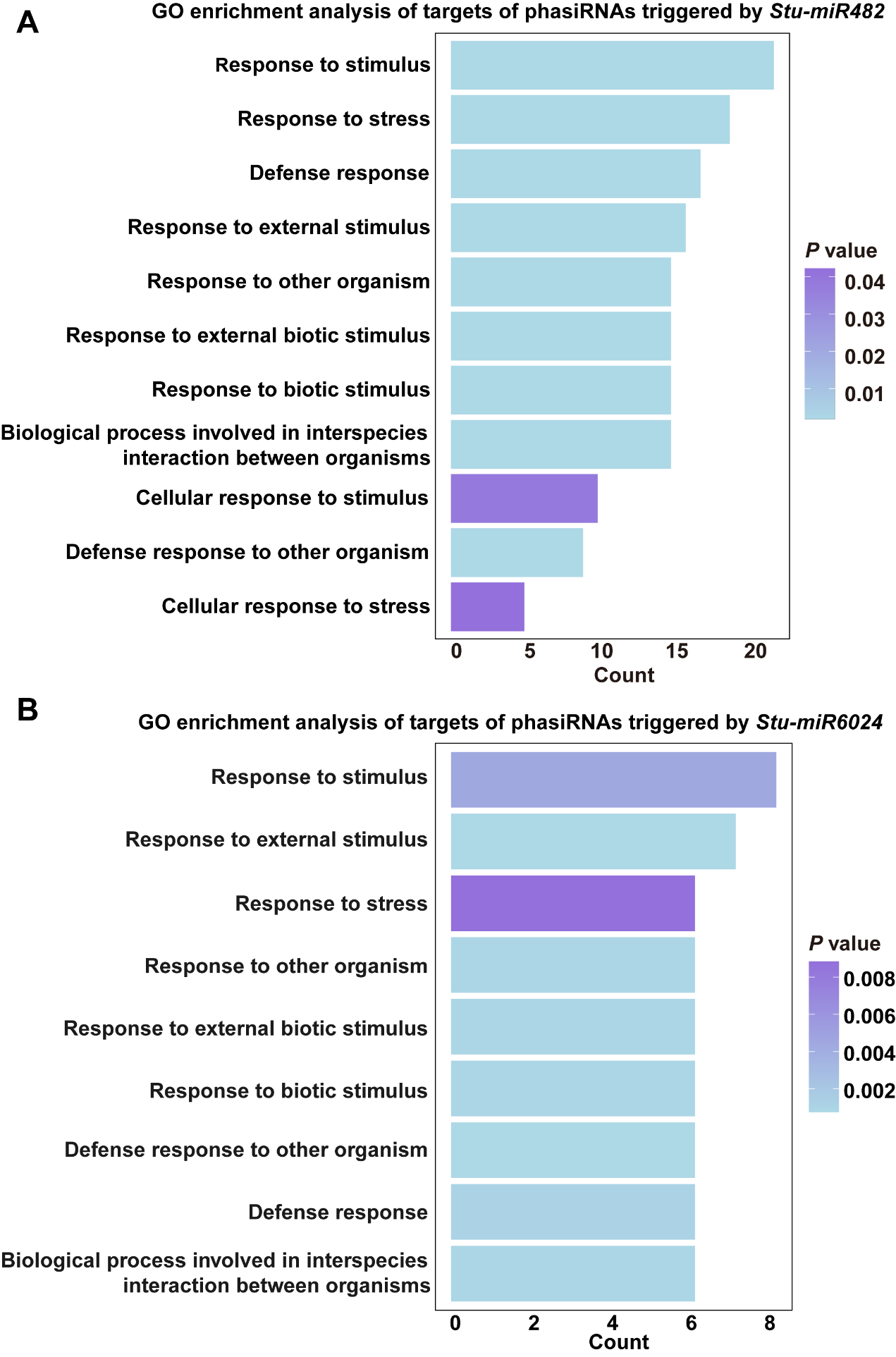


**Supplementary Figure S10. GO enrichment analysis of genes targeted by *Stu-miR482* and *Stu-miR6024*-triggered phasiRNAs in potato.**

(**A-B**) GO enrichment analysis of target genes of *Stu-miR482* (**A**) and *Stu-miR6024* (**B**)-triggered phasiRNAs. The horizontal axis represents the count of enriched genes, and the vertical axis represents the enriched GO terms (**A-B**).
